# Ozone Treatments for Preserving Fresh Vegetables Quality: A Critical Review

**DOI:** 10.3390/foods10030605

**Published:** 2021-03-12

**Authors:** Elodie Sarron, Pascale Gadonna-Widehem, Thierry Aussenac

**Affiliations:** Institut Polytechnique UniLaSalle, Université d’Artois, ULR 7519, 19 Rue Pierre Waguet, BP 30313, 60026 Beauvais, France; elodie.sarron@unilasalle.fr (E.S.); gadonna.pascale@gmail.com (P.G.-W.)

**Keywords:** ozone, food preservation, fresh vegetables, food industry, food quality, food safety, microorganisms, shelf-life

## Abstract

Ozone is recognized as an antimicrobial agent for vegetables storage, washing, and processing. This strong disinfectant is now being used in the food industry. In this review, the chemical and physical properties of ozone, its generation, and factors affecting ozone processing efficiency were explained as well as recent regulatory developments in the food industry. By then selecting three vegetables, we show that ozone avoids and controls biological growth on vegetables, keeping their attractive appearance and sensorial qualities, assuring nutritional characteristics’ retention and maintaining and increasing the shelf-life. In liquid solution, ozone can be used to disinfect processing water and vegetables, and in gaseous form, ozone helps to sanitize and preserve vegetables during storage. The multifunctionality of ozone makes it a promising food processing agent. However, if ozone is improperly used, it causes some deleterious effects on products, such as losses in their sensory quality. For an effective and a safe use of ozone, specific treatment conditions should be determined for all kinds of vegetables. In a last step, we propose highlighting the different essential characteristics of ozone treatment in order to internationally harmonize the data relating to the treatments carried-out.

## 1. Introduction

Nowadays, vegetables represent an important part of the daily diet and a considerable segment of the food market. Indeed, due to their nutritional value, they are indispensable for a healthy and balanced diet (i.e., low content in fat, sugars, and sodium). Moreover, vegetables are rich sources of vitamins, minerals, dietary fibers, complex carbohydrates, and non-nutrient substances including plant sterols, flavanols, anthocyanins, and phenolic acids. Eating a wide variety of vegetables helps to ensure an adequate intake of essential nutrients, and that is why the World Health Organization (WHO) suggests that everyone should consume a minimum of 400 g of fruits and vegetables daily as a way to improve overall health [1]. This consumption reduces the risk of certain nonchronic diseases including certain types of cancer and cardiovascular diseases; also, it prevents weight gain and reduces the risk of obesity [2]. Vegetables are also well-appreciated due to their attractive sensorial qualities as well as their taste, aroma, texture, color, gloss, shape, size and, absence of defects and decay. Eighty percent of purchasers pay a lot of attention to the appearance of these products [3]. Qualitative criterion appears to be the main criterion of choice [4]. However, their short shelf-life is associated with a large number of foodborne illness outbreaks that have been implicated with their consumption [5]. This identifies the great importance of applying adapted treatments in order to decontaminate vegetables efficiently and/or avoid microbial development.

With the aim of extending the shelf life of vegetables, conventional chemical treatments, described as antimicrobial solutions, are usually applied: chlorine, peracetic acid, electrolyzed water, hydrogen peroxide, etc. The first one, sodium hypochlorite (chlorine), has been the one most routinely used by the food industry in aqueous formulations under different conditions (washing, spraying) in recent decades. Chlorinated water is also the chosen treatment selected as a reference in the majority of scientific works whose aim is to find its effective alternative. It has been demonstrated that chlorine acts effectively on foodborne pathogens [6] while maintaining the overall quality of the treated product during its shelf-life [7,8]. However, consumers have also become more critical of the use of synthetic additives as their awareness of health and food safety has increased [9]. Some European countries have forbidden the use of chlorine because of its reaction with organic matter, bromide, and iodide to form hazardous chemicals in wastewater such as brominated and iodinated disinfection byproducts, monochloramine, organochlorinated byproducts, halo acetic acids, and trihalomethanes, [10,11]. These byproducts are cytotoxic to mammalian cells, genotoxic with induction of DNA damage, mutagenic, and persistent in the environment [11,12]. Due to these drawbacks and the rising demand for natural additives, the development and application of more green technologies for preserving vegetable safety and quality have always been industrial concerns. In this context and from available technologies, ozone application is promising, and it is gaining interest in the vegetables industry [13].

Ozone (O_3_) is a powerful sanitizer that may meet the acceptance of consumers, the expectations of manufacturers, and the approval of regulatory agencies. Ozone was Generally Recognized As Safe (GRAS) in 1995 in the USA for the disinfection of bottled water. Since 1997, ozone has GRAS status for direct contact with foods. In June 2001, ozone, in gas and aqueous phases, was approved by the US Food and Drug Administration (FDA) as an antimicrobial additive for direct contact with foods. This was done in response to an Electric Power Research Institute (EPRI) food additive petition [14]. In the European Union, application of ozone in food processing commenced in the early 1900s after its first use for water treatment. The European Council of Ministers has adopted a proposal which permits the ozone treatment of natural mineral water. In France, during 2003 and 2004, the French Food Safety Authority (AFSSA now called ANSES) rendered two opinions regarding the safety of using ozone as an auxiliary technology to treat wheat grains before grinding. The use of ozone has been authorized by the regulatory agency since 2006 as a processing aid for flour quality improvement, based on treatment by ozone in a closed sequential batch reactor [15]. In 2019, ANSES rendered an opinion to extend the use of ozone in water, as a technological aid, for the washing of ready-to-use salads [16].

Since then, research and commercial applications have been conducted in order to confirm that ozone can replace traditional sanitizing agents and provide benefits for obtaining safe products with extended shelf life. Additionally, possible uses and the beneficial or detrimental effects of ozone have been intensively investigated for various vegetables. In the vegetable handling process, ozone can be applied in two forms. Gaseous ozone is added continuously or intermittently to the storage atmosphere of the harvested product. Aqueous ozone is added immediately after the vegetable harvest or during the washing treatment. In this latter case, the product can be washed in water containing dissolved ozone by spraying, rinsing, or dipping. The application of ozone in the vegetables industry has been reviewed already [13,17,18,19,20,21]. Nevertheless, this review intends to collect and summarize all of the recent studies that are not covered in previous works on certain vegetables produced and consumed around the world. We have chosen three different vegetables: a root vegetable (carrot), a green leafy vegetable (lettuce), and a fruiting vegetable (tomato). These three vegetables have been chosen for several reasons: not only are they widely consumed worldwide, but they also have different characteristics due to the fact that they are grown in varied conditions. For these reasons, we will approach the effects of ozone on microbial, sensorial, and nutritional quality and also on the physical and chemical properties of these vegetables. The originality of the proposed approach is that it deals with the overall quality of vegetables (microbial quality, physical and chemical quality, as well as nutritional quality) immediately after the washing procedure and during the storage.

## 2. Use of Ozone in Vegetables Industry

Ozone is triatomic oxygen that naturally occurs in the earth’s atmosphere where it is found in gaseous form and in very low concentrations. In the stratosphere, it results from the photodissociation of dioxygen molecules under the action of solar radiation. This phenomenon leads to the formation of the ozone layer, which represents almost 90% of the total atmospheric ozone and protects terrestrial organisms against harmful UV radiation (characterized by wavelengths between 200 and 300 nm) from the sun. At the same time, ozone degrades organic matter in the lower layers of the atmosphere and impairs the proper functioning of living organisms; among other things, it is responsible for respiratory diseases. This ambivalence makes it a very remarkable molecule.

### 2.1. Physico-Chemical Properties of Ozone

**Figure 1:**
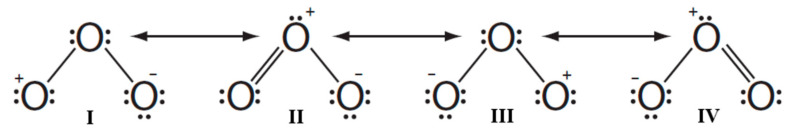
Electronic structure of ozone [22].

Ozone is an “angled” molecule. It is in the form of an isosceles triangle with an apex angle equal to 116°49′. The two interatomic O‒O bonds have a length of 127.8 µm. The molecule can be thought of as a resonant hybrid of four mesomeric forms that form the basis of ozone chemistry. The central atom of the four mesomeric forms is sp² hybridized and has an entire byte of electrons. According to the literature, theoretical calculations show that there is a 50% probability that the bond between two oxygen atoms is a double bond (Figure 1). So, the electronic structures II and IV essentially represent the electronic structure of ozone. However, the resonance forms I and III also contribute to a certain extent to the ozone molecule; in particular, the value of the angle at the top is due to the attraction of the terminal atoms which are charged positively and negatively, respectively.

This structure gives this molecule a metastable character under ambient conditions. Ozone has a natural tendency to break down into dioxygen (O_2_) and atomic oxygen (O) or to react with other compounds. This ability to easily give up an oxygen atom gives it a very strong oxidizing power (E^0^ = 2.07 V) [23] compared to chlorine (E^0^ = 1.36 V) and oxygen (E^0^ = 1.23 V). At room temperature and at atmospheric pressure, ozone is a colorless gas, but it has a bluish appearance when it is present in high concentrations. Ozone liquefies at −111.35 °C in the form of a dark blue liquid, and it solidifies into a dark purple solid at −192.5 °C. Ozone has a characteristic, penetrating and pungent odor, which is quickly detectable. The odor perception threshold is set at 0.01 ppm. The main physicochemical properties of ozone are synthetized in Table 1.

### 2.2. Ozone Generation

The so-called “technical” ozone is produced artificially to be used as an oxidant. Since ozone is a very unstable gas which decomposes very quickly into diatomic oxygen, it must be produced in situ at the place of use. Therefore, it cannot be stored or transported like other industrial gases. The overall reaction involved in ozone generation is endothermic (requires energy such as heat) [24].

(1) $$3O_{2}⇌2O_{3}\;{\Delta H}^{0}=+142.2\;{\mathrm{kJ}\;\mathrm{mol}}^{-1}\;\left(\mathrm{at}\;1.013\;\times 10^{5}\;\mathrm{Pa}\right),{\;\Delta S}^{0}=-69.9{\;\mathrm{kJ}\;\mathrm{mol}}^{-1}K^{-1}$$

This energy can only be supplied by

○ Electrolyzing water,
○ Photolyzing the oxygen by irradiating it using UV at wavelength lower than 220 nm,
○ Using ionizing irradiation to radiolysis the oxygen,
○ A high voltage electrical discharge into the oxygen stream.

**Figure 2:**
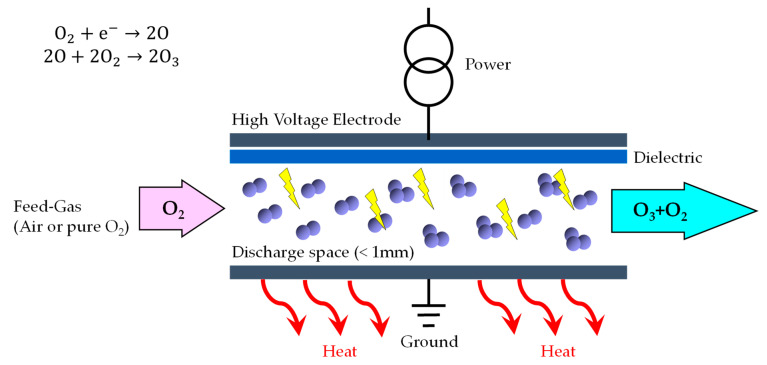
Ozone production from corona discharge.

The latter process, using a corona discharge (CD), is widely used in industry. This principle is as follows: a flow of dry gas containing oxygen, called gas vector, flows through a thin gap separating two metal electrodes; at least one of the electrodes is covered by a dielectric material (Figure 2). Alternative tensions of a few thousand volts are applied between the two electrodes, either at the network frequency or an average frequency. These voltages generate filamentary electric discharges between the two electrodes (low intensity streamers) which dissociate the gas and generate an unstable and very strongly ionized called “cold plasma”. Oxygen molecules (O_2_) are split in the gas, resulting in the formation of oxygen atoms (O). Seeking stability, these attach to other oxygen molecules (O_2_) to form ozone (O_3_).

By the CD process, the production of 1 kg of ozone at the mass concentration of 12% in oxygen, requires the use of 8.3 kg of oxygen and the consumption of 10 kWh of electricity. This power consumption is very dependent on the concentration of ozone in its carrier gas. The higher the concentration of ozone in the carrier gas, the higher the consumption of electrical energy.

At the heart of a corona discharge ozone system is the dielectric. The electrical charge is diffused over this dielectric surface, creating an electrical field, or “Corona”. Proper air preparation is critical to CD ozone systems. The gas feeding the ozone generator must be very dry (minimum −70 °C) because the presence of moisture affects ozone production and leads to the formation of nitric acid which is very corrosive to critical internal parts of a CD ozone generator (i.e., this can cause premature failure and will significantly increase the frequency of maintenance). Since 85% to 95% of the electrical energy supplied to a corona discharge ozone generator produces heat, some method for heat removal is required. In addition, proper cooling significantly affects the energy efficiency of the ozone generator, so most corona discharge systems utilize one or more cooling methods (air and/or water).

### 2.3. Transfer and Monitoring Ozone in Air and Water

Once ozone gas has been produced, the next step is to apply it, either in gas form or by dissolving it in water. As we will see later in this article, these two forms of ozone preparation (i.e., gas and liquid) are commonly used in the field of fresh vegetables. Whatever the form of ozone used, good control of the transfer of this molecule to the target medium is decisive because it determines the effective ozone concentration profile which has a considerable effect on ozone consumption, reaction kinetics, sanitizing power, etc. Moreover, it is important to ensure the highest efficiency and lowest residual ozone mass due to the high costs associated to ozone generation. The main parameters controlling the transfer of ozone are listed below:

- Concentration of ozone in the carrier gas
  ○ Ozone application pressure (liquid height, pressurized gaseous sky)
  ○ Size and rate of rise of bubbles
  ○ Hydrodynamics at the gas—liquid interface (periphery of the bubbles)
  ○ Temperature and pH of the solution
- Solid phase transfer
  ○ Structure of the solid (surface state)
  ○ Surface/volume ratio (particle size)
  ○ Physical structure of its periphery accessible to gas
  ○ Chemical composition of the solid (reactivity)
  ○ Water activity of the solid

Regardless of the ozone application methodology chosen, it is essential to be able to measure and monitor the ozone concentration used during the process in order to be able to define the quantities strictly necessary to ensure adequate efficiency for the disinfection of a solution and/or a solid matrix. The main methods used to measure and monitor the ozone concentration are listed below:

- Quantification of dissolved ozone in water solution

The quantification of ozone in solution can generally be done by two ways—namely, by volumetry and/or spectrophotometry. Between these procedures, the most common method used for analysis of ozone is the volumetric method (i.e., iodometric titration) even if it is more laborious [25]. At the same time, different spectrophotometric methods exist. They all use a particular compound which reacts stoichiometrically with ozone (i.e., potassium iodide for the DDPD procedure, indigo trisulfonate for the indigo procedure, and methyl orange for a new procedure) and whose transformation can be evaluated by spectrophotometric absorption under ultraviolet and visible light [26,27]. Finally, continuous measurement of dissolved ozone can be performed by amperometry using measuring cells which consist of a gas-permeable membrane stretched tightly over a gold cathode completing a circuit represented by a silver anode and an electrolyte solution [28].

- Quantification of ozone in air

Even if chemiluminescence and UV-absorption based methods have been used in the past 20 years for the measurement of ozone in ambient air, it is now generally accepted to use UV-photometry (253.7 nm) as the primary quantification/calibration method. Since the UV-absorption method has proven to be reliable and robust in field operations, this method is the one most often recommended, and its use follows the principles of the International Organization for Standardization (ISO) [29].

### 2.4. Factors Affecting Ozone Processing Efficiency

The efficacy of ozone is affected by both intrinsic and extrinsic parameters, and it is difficult to predict ozone behavior on fruits and vegetables in the presence of specific compounds like organic matter and environmental factors. In addition, parameters and factors influencing the efficiency of ozone treatment are mentioned in Table 2 from O’Donnell et al. [28] and completed with bibliographical research.

#### 2.4.1. Extrinsic Parameters

Since ozone is unstable in an aqueous solution or in air, its effectiveness as a disinfectant depends not only on the amount used but also on the residual ozone in the medium and various environmental factors such as medium pH, temperature, humidity, and the amount of organic water surrounding the product and microorganisms [30].

To ensure a high level of microbial destruction by ozone, the decomposition rate of ozone must be as low as possible in the treatment environment. The pH affects the ozone decomposition reaction.

Under acidic conditions (pH around 3.0–4.0), ozone was found to be reasonably stable, and its decomposition rate was found to be relatively slow [31,32]. With increasing the pH (but always under or around 7.0), ozone degradation is accelerated due to hydroxyl radical formation, which is the main cause of ozone decomposition. Under alkaline conditions (pH around 9.0 and more), the importance of the peroxy-radicals and the hydroxide ion initiation step increases and accelerates ozone decomposition [33,34,35]. Consequently, it was observed that microbial killing by ozone was much faster at lower pH, and survival is better at pH greater than or equal to 7.0.

The ozone treatment duration required for achieving a 5 log CFU (Colony-Forming Unit) mL^−1^ reduction of two *Escherichia coli* strains in apple juice was faster (4 min) at pH 3.0 than at pH 5.0 (18 min) [36]. Moreover, the survival of *E. coli* was higher at pH 8.0 as compared to lower pH values in various types of water [37]. The optimum buffer pH for *Staphylococcus aureus* survival is 5.5 to 6.0, in comparison with lower pH [38]. As the pH has a considerable effect on the percentage of disinfection by hydroxyl radical production initiated by the ozone chain reaction, it is also important to consider the concentration of organic matter.

An increased ozone demand can be caused by suspended solids which can be organic or inorganic. An organic load present during treatment is known to decrease the effectiveness of ozone for the inactivation of microorganisms by consuming ozone [39]. In the study conducted by Hunt et al. [40], the rate for *E. coli* inactivation by ozone in the presence of humic acid was slower than in the absence of natural organic matter. This was due to the faster decomposition of dissolved ozone in presence of organic matter, and consequently the lower exposure of *E. coli* cells to this disinfectant. In addition, a faster inactivation rate of *E. coli* was achieved in a model orange juice solution (1 min) in comparison with a juice with low pulp content (6 min). Whereas, the inactivation in unfiltered juice was after 15 to 18 min [41]. These results indicated that organic matter significantly interferes with the antibacterial activity of gaseous and aqueous ozone. Moreover, Restaino et al. [30] found that the type of organic matter affects ozone effectiveness more than the amount of organic materials present in the suspension. They reported that in ozonated water containing organic material, death rates of tested bacteria (*S. aureus*, *Listeria monocytogenes*, *E. coli* and *Salmonella typhimurium*) were not significantly affected by addition of 20 ppm of soluble starch but were significantly reduced by addition of 20 ppm of bovine serum albumin [30].

Factors influencing the solubility, stability, and reactivity of ozone may also affect the efficacy of ozone. The temperature affects the biocidal efficiency of ozone. A reduction in the temperature of an aqueous medium increases ozone solubility and stability, increasing its availability in the medium and, consequently, increasing its efficacy [28]. The inactivating capabilities of ozone are correlated with decreasing temperature. As the temperature increases, ozone becomes less soluble and less stable with an increase of its decomposition rate [42].

Regardless of the ozone form, ozone concentration and treatment time are two extrinsic parameters determining ozone efficiency. This efficacy on a target microorganism is described by the CT concept where C refers to the residual concentration of ozone in mg L^−1^ and T refers to the contact time in minutes. Therefore, the intensity of an ozone treatment is expressed in terms of CT (mg min^−1^ L^−1^) coupled with the target microorganism and the surrounding conditions. In most cases and for the same CT value, a low ozone concentration combined with a high treatment time is equivalent to the combination of a high ozone concentration and lower treatment time [43,44]. However, such equivalence has been proven invalid in certain cases where high concentrations applied over short time intervals were shown to be more phytotoxic than identical exposures in which lower concentrations were applied over longer time intervals [45]. Moreover, Finch et al. [46] indicated that the method of determining CT by using the final concentration of reactants at the end of the contact time overestimates the CT needed for disinfection.

The application of aqueous ozone in two different modalities (static or dynamic) had different antimicrobial effects. Indeed, the rate of destruction of the attached bacterial cells is higher in the dynamic conditions than static ones, regardless of the microbial species [47]. Bubbling ozone in water containing a shredded lettuce mixture was more efficient with high-speed stirring than at low-speed [48]. In addition, sanitization treatment of apples artificially contaminated with *E. coli* was more effective when ozone was bubbled during apple washing than by dipping apples in preozonated water [49]. Moreover, the bubble size in the water is also crucial in studying the effectiveness of the disinfection treatment. For a given concentration of ozone at a constant gas flow rate, a decrease in bubble sizes from 2.38 mm to 1.72 mm resulted in an increase in residual ozone and microorganism inactivation [50]. Bubbles with a diameter of 0.1 cm have nearly 32 times more contact value than those of 1.0 cm diameter [51].

It has been reported in scientific literature that gaseous ozone is a less effective antimicrobial agent than aqueous ozone [47,48]. It has been widely reported that a high relative humidity is needed for inactivation of microorganisms by ozone gas. The optimum relative humidity of a gas is about 90 to 95%, and ozone loses its bactericidal efficiency at 50% or below [52]. The strong effectiveness of ozone gas at high relative humidity levels is beneficial for sanitation of fruits and vegetables where environmental relative humidity is generally more than 80% [53]. This moderate effect exerted by gaseous ozone is strictly due to the mechanism of action of the ozone, which requires the presence of water, and theoretically, an increase in the relative humidity of the gas intensifies the efficiency of gaseous ozone [47].

Although ozone efficiency is widely affected by extrinsic factors, authors should not overlook the high importance of intrinsic factors.

#### 2.4.2. Intrinsic Parameters

As listed in Table 1, the effectiveness of ozone for decontamination can be affected by intrinsic characteristics of vegetables and their microbial population.

The microorganism type, physiological state, concentration, and stress significantly influences the antimicrobial impact of ozone [21,54,55]. The type of microorganism as well as the age of cells can impact its susceptibility to ozone inactivation [56]. Wani et al. [55] observed that for cells of *Pseudomonas* spp., older colonies (7, 10, and 12 days old) were more resistant to gaseous ozone than cells from younger colonies (2 and 4 days old). Moreover, these authors observed that *Pseudomonas* sp. submitted to refrigerated conditions show enhanced resistance to ozone in vitro. Ozone provokes bacterial aggregation and noncultivability of *P. syringae* prior to viability loss [57]. Microorganisms embedded in surface irregularities are more protected from ozone than those readily exposed [21]. For example, after an artificial contamination of lettuce leaves by *E. coli*, inactivation by chlorine treatment was most effective for the intact leaf surface than for trichrome, stomata, and cut edges of damaged lettuce leaves [58]. Kroupitski et al. [54] also observed that the effect of the sanitizer was significantly greater on intact tissue than on cut tissue artificially contaminated with *Salmonella*. These cells had attached to the cuticle of the intact leaf surface, while the majority of cells were located in the cut-edge regions, with a preference for the wounded tissue. By using stained bacteria, Wani et al. [55] mentioned that bacteria formed large aggregates which were preferentially attached to the epidermal cell margin. Microcolonies and biofilms were formed on leaf surfaces due to bacterial attachment and production of exopolymeric substances. Confocal images of ozone treated leaves also revealed that two and three live cells survived in microcolonies surrounded by dead cells [55]. However, individual surviving bacteria constituted 10% of the bacterial viable counts on the leaf surface [55]. Therefore, cells in microcolonies and biofilms on leaf surfaces may resist ozone treatment by both physical protection and by the biofilm bacteria themselves having enhanced resistance mechanisms [55,59].

The aw of the product is also a significant parameter related to the efficiency of ozone treatment. Kim et al. [21] treated a powdered food-grade ingredient with variable aw with gaseous ozone at 200 ppm. When the aw of the product was 0.95, more than 2 log CFU/g were inactivated. However, an aw less than 0.84 had no effect on the microbial load of products at a similar ozone concentration. When the aw of the product was increased from 0.84 to 0.95, ozone was as effective in decreasing the microbial load because it was in the product that naturally contained a high aw [60]. Sarron et al. [61] observed no significant effect of gaseous ozone concentration on fresh and lyophilized *G. stearothermophilus* spores stored at different aw between 0.06 and 0.98 in any tested ozone concentrations for 30 min. Moreover, an aqueous treatment (aw = 1) at 3.8 g/Nm^3^ of a spore suspension reduced spore counts by 5.5 CFU/mL in 25 min. Inactivation of food microorganisms by ozone significantly depends on the food surface (nature, chemical composition, texture), the microorganisms characteristics (type, contamination load and the degree of attachment).Application of aqueous ozone produced promising results with a low ozone demand for products having smooth and intact surfaces, such as apples [49], tomatoes [62], and green peppers [63]. They represent easy products to allow direct contact of the sanitizer with the bacteria. Microbes should be easily detached from plant tissue. For example, an apple’s surface is smooth and regular and easily exposed to ozone, in comparison with the stem-calyx region which is irregular with places for microbes to hide [49]. However, contradictorily to Kim et al., research by [48] Alexopoulos demonstrated in 2013 [63] that lettuce has an extremely irregular and rugged surface with many hides places which could be a niche for bacteria.

When the surface is more complex in terms of porosity and roughness, on carrot roots for example, the microbial inactivation seems to be more complicated [62]. The inactivation of microorganisms is greatly affected by the porous surface of carrots, which allows bacteria to be protected against ozone treatment [64]. Consequently, it is important to ensure direct contact between ozone and the target microorganisms. In this context, a variety of application methods are employed including washing, dipping, stirring, and bubbling in order to increase the quality of treated products.

Ozone destroys microorganisms by the progressive oxidation of vital cellular components. The bacterial cell surface is the primary target of ozone and formation of ruptures in the cell wall with consequent cellular disintegration that can occur as a result of the oxidation process. At the beginning of ozone treatment, Gram negative bacteria such as *E. coli* are more susceptible to ozone because of their thin peptidoglycan lamella which is covered by an outer membrane made of lipoproteins and polysaccharides. The D-values were higher for Gram positive compared to Gram negative bacteria. When the ozone treatment was prolonged, bacterial destruction was strain-related rather than Gram-related [63,64].

After describing the use of ozone in the vegetable industry, we will now focus on describing the role and impacts of gaseous and aqueous treatments with ozone on three different food matrices: fresh carrots, lettuce and salads, and tomatoes.

## 3. Effects of Ozone Treatment on Carrot Quality

Carrots are one of the ten most economically important vegetables crops grown throughout the world [65]. In 2012, almost 37 million tons of carrots were produced worldwide for human consumption. About 61.8% of the world carrot production occurred in Asia, followed by Europe (22.6%) and America (9.1%). Orange colored carrots are predominant around the world. Most of the taproot consists of a pulpy outer cortex (phloem) and an inner core (xylem). High quality carrots have a large proportion of cortex compared to the core. Carrots contain a variety of biologically active substances like carotenoids, dietary fibers, and vitamins. The consumption of fresh carrots is increasing due to its recognition as an important source of natural hydrophilic and lipophilic antioxidants such as chlorogenic acids, lutein, and lycopene, which have anticancer activity [66]. A high initial total viable count of 5–6 log CFU g^−1^ associated with low-acid conditions (pH 6.0–6.5) is advantageous to allow a rapid increase in the microbial population. Therefore, fresh carrots, which have a short shelf life, should be consumed within a few days, limiting their market potential and also leading to microbiological safety problems. However, under ideal storage conditions of 0 °C and high relative humidity (98–100%), fresh carrots can be stored for up to five months [67]. Nevertheless, carrot quality may decline with excessive decay caused by microorganisms like *Sclerotinia sclerotiorum* and *Botrytis cinerea*, the development of bitterness, and the loss of texture and flavor. In this context, ozone is the technology which has the potential, firstly, to reduce the decay during a long storage period while maintaining quality of stored carrots and, secondly, to increase shelf life of fresh carrots found in the marketplace after a step to reduce microorganisms during washing.

### 3.1. Effect of Continuous Gaseous Ozone Exposure on the Quality of Stored Carrots

The objective of a continuous ozone treatment is to increase the storage time and the shelf life of fresh whole carrots just after their harvest. A compilation of research studies is presented in Table 3, with emphasis on the ozone impact on microbial inactivation and quality aspects of fresh carrots.

First of all, we will focus on the visual aspect of carrots, which is, as mentioned before, the first criterion for consumer purchases. The effect of ozone on quality parameters, such as color, was described in all studies. The visual quality of the product is important because any color alteration might be recognized as a symptom of senescence. No significant change of carrot color was shown after a treatment at 450 ppb (0.45 mg L^−1^) for 48 h [69], 7.6 mg L^−1^ for 15 min [72], and between 1 to 5 mg L^−1^ for 9.5 to 110.5 min [74]. However, the ozone treatment caused some injury which appeared as dry white blotches at 60 µL L^−1^, 8 h daily, for 28 days [68], scattered blotches of slightly brown discolored periderm at 50 nL L^−1^ for 6 months [71] and bleaching [73]. These deleterious effects indicate that carrots suffered significant physiological injury as a result of the oxidative stress produced by ozone. The white discoloration of carrots is the product of dehydration of the surface. [75]. Further, alterations in appearance of ozone-treated carrots due to color changes and surface pitting may affect consumer appeal.

In most cases, ozone treatment did not affect physical and physiological carrot quality immediately after the treatment and during the storage period for the tested concentration and treatment period in most published studies [71,74]. However, some authors have demonstrated a negative effect of ozone on fresh carrots, which we will describe one by one. Firmness associated with weight loss is an important rheological property which is pertinent for fresh carrots. Carrots that have a firm texture is a sign of freshness and wholesomeness. Many studies showed that ozone did not have any effect on the firmness of carrots [71,74]. On the other hand, in response to ozone treatment, a delay of tissue toughening and a reduction of firmness was observed by Forney et al. [67]. This was associated with changes in cellulose, lignin, and hemicellulose content, which was due to the reduced lignification of cell walls [70,76].

Moreover, when gaseous ozone is used in postharvest treatment during storage, its high oxidation power promotes other undesirable changes in carrot quality. Symptoms of physiological disruptions included an increase of respiration rates, electrolyte leakage, and sucrose concentration [68,70]. The increase of terpenes and hexanal in the headspace indicates the occurrence of lipid oxidation and suggests that this treatment may enhance carrot flavor [70]. Ozone acts as a postharvest stress which stimulates respiration and ethanol production [70]. These higher respiration rates are the consequence of an abnormal metabolism caused by an increase of ozone concentration [68]. An ozone supply of 15 µL L^−1^ for 8 h a day for 28 days provides some disease protection with a minimum of physical and physiological damage [68].

Concerning the microbiological quality of carrots, most studies concluded that an increase of bactericidal effect is correlated with an increase of ozone concentration and exposure time [72,73]. However, ozone treatment at an increased concentration does prejudice the initial quality of carrots, regarding its color characteristics. The effect of ozone is on the outer surface of the roots and it would therefore involve an inhibition of microorganisms which are mostly located on the surface of the core. This surface treatment also involve a physical alteration of the carrots’ surface, which could result in insignificant variations in the pH, soluble solids (SS), glucose, fructose, sucrose, and galactose of the carrot. Moreover, ozone has increased the shelf-life of carrots [74]. Ozone on *B. cinerea* and *S. sclerotiorum* was fungistatic and not fungicidal [68,69,70,71]. The treatment involved an increase of isocoumarin concentration, which contributed to the reduced rate of lesion expansion caused by these two alteration microorganisms [71]. Moreover, gaseous ozone had a bactericidal effect on *E. coli* O157:H7 [72], Shiga toxin-producing *E. coli* (STEC), *Salmonella enterica*, and *Listeria monocytogenes* [73] that increased with concentration and length of exposure.

We have described the effectiveness of ozone on the visual, sensory, and nutritional quality of carrots. Now, we must focus on the processing conditions of carrots.

All authors indicated the ozone concentration in the gas measured at the beginning of the treatment when it penetrates in the reactor where the carrots are located. Liew et al. [68] reported that applying an ozone concentration of 60 µL L^−1^ resulted in a different residual ozone concentration, from 22 to 18 µL L^−1^ at 2 and 16 °C and identified that residual ozone concentration is influenced by temperature and applied ozone concentration. Indeed, they measured a higher residual ozone concentration at 2 and 8 °C than at 16 °C. Moreover, a significant difference was observed between the application concentration and the residual concentration. It is essential to indicate the ozone concentration in the reactor at the beginning, during, and at the end of the treatment in order to know the loss of ozone caused by different phenomenon like dilution in the volume of the treatment container, reaction with the plant compounds such as pesticide residues or microorganisms, the purge duration, etc.

It is important to note that the units used by the authors are not homogeneous. In addition, the unit used in the work of Liew et al. [68] was µL L^−1^. In this study, the ozone concentration was monitored with an ambient ozone analyzer model IN-2000-5 using UV absorption, which is supposed to indicate ozone concentrations in ppm from 0 to 1000 ppm, according to its operating and maintenance instructions manual [77]. A similar unit (nL L^−1^) was used by Forney et al. [70] and Hildebrand et al. [71] who treated carrots with the same generator sold by Simpson environmental Corporation. However, Sharpe et al. [69] indicated that this generator gave an ozone concentration in ppb in carrot treatment. The unit “ppb” or “ppm” means “parts per billion” (10^−9^) or “parts per million” (10^−6^) and represents a ratio and no indication specifies if it is a volume, mass, molar concentration, or a massic concentration. International units should be used by authors. Moreover, a real harmonization of the units is fundamental in order to compare effectively the different treatments and to allow the reader to reproduce the whole treatment in the same conditions. Bridges at al. [73] presented the ozone concentration characteristics in mg/m^3^ and also in a processing rate expressed in µg O_3_ g^−1^ of produce to make similar working units. This additional information indicated that the treatment doses of 0.86 and 1.71 µg O_3_ g^−1^ made the comparison easier between several scientific works. However, this processing rate cannot be calculated in all scientific articles due to the significant lack of information indicated in scientific publications (e.g., absence of exact quantity of treated product, no specified flow rate, etc.).

As we have just seen, there is great variability between the ozone concentrations, time duration, application method, and environmental conditions in terms of pH, temperature, and humidity applied, and the effects of ozone on the quality of carrots are all the more variable, which have a real impact on the carrot’s quality, as presented in Table 4.

As presented in the Table 4, although the variability of the treatments is very high (e.g., in terms of CT, it comprised between 1.73 and 804.6 mg min^−1^ L^−1^), all of these conditions are quite effective and involved a relative conservation of visual and physical qualities of stored carrots and a significant increase of microbial quality. However, further studies are needed to supply the optimum ozone concentration in the optimum conditions (time, RH, temperature, flow rate, etc.) to control decay and maintain quality with a minimum of physical and physiological damage. Moreover, we observe upon reading Table 4 a significant lack of information concerning the impact of the ozone treatment on the nutritional quality of the stored carrots. This could be an area of interest for future works.

### 3.2. Effect of Ozone Exposure during Washing on the Quality of Carrots

The objective of this treatment is to wash and decontaminate carrots in order to market them immediately. An assembly of research is presented in Table 5, with emphasis on the impact of ozone on microbial inactivation and quality aspects of freshly washed carrots.

Among these scientific publications, two authors applied ozone after a first washing with tap water in two different conditions: spraying ozonated water for 2 min [78] or on a bed reactor inside a chamber [62]. In the other works, ozonated water was prepared in a reservoir by circulating potable tap water or deionized water through an ozone generator using oxygen or air as feed gas [79,80] or by bubbling ozone gas into water [72,74,76]. In these cases, the indicated concentrations are those measured by the analyzer at the end of the preparation of the washing water and before the step of the treatment beginning [72,76,79]. Although, the fact that the ozone solution was used within 2 min of removing it from the gas [72] or used immediately after the desired ozone concentration was reached [79], the ozone concentration in the water necessarily decreased with the washing. However, few researchers have focused on the ozone concentration in real time during the treatment and at the end of the washing time. For example, Alegria et al. [79] prepared 1 ppm ozonated water. During the 5 min washing time, the concentration decreased to 0.08 mg L^−1^ min^−1^ for precut carrots and 0.32 mg L^−1^ min^−1^ for postcut carrots. In comparison, the concentration decreased more rapidly with postcut samples because of the higher amount of organic matter in the washing water caused by highest leaching rates on these shredded carrots. This phenomenon can be explained by a larger surface area exposed to the immersion treatment which contributed to the reduction of ozone availability.

Firstly, no color changes were observed in fresh entire carrots treated with ozonated water [64,72,74]. Color changes were minimized for precut carrots [79] or carrots sticks [76]. Alegria et al. [79] showed significant color changes in washed carrots and particularly for shredded carrots. The color value of shredded carrots was significantly influenced by the washing treatment because of the intense leaching phenomenon. In another study, similar results were observed with an increase in the whiteness index [62]. Whiteness is recognized as a particular attribute of color and many studies were carried out in order to define whiteness in a colorimetric way, based on CIELAB measurements. In published studies white appearance is considered a result of either surface dehydration of outer layers or enzymatic activity and the formation of lignin. This leaching phenomenon observed for shredded carrots is also the source of a considerable loss of solid soluble content (soluble sugars and aroma) which could affect the sweet-taste perception of carrots [79]. This phenomenon was not observed for whole fresh carrots [64]. In the study of Souza et al. [74], it was verified that the temperature associated with the ozone concentration could affect the pH of carrots. An increase of the ozone concentration to above 5 mg L^−1^ prevented immediate changes in the pH of carrots when they were exposed to ozonated water above 14 °C. However, throughout the storage time, the pH of carrots did not change and values were similar to the expected ones for harvested carrots during storage [74,79]. This finding suggested that the ozone concentration and its interaction with water temperature temporarily affect the pH of carrots. Ozone was found to decrease the respiration and ethylene emission rate which are crucial as metabolic and tissue senescence determine the overall keeping quality during 30 days of storage [76].

Secondly, concerning microbiological quality, all tested liquid ozone treatments achieved a significant reduction in total mesophilic aerobic bacteria [64,76,79,80], yeasts and molds [64,79,80], coliforms [64], *E. coli* [62,64,72], and *Salmonella enteridis* [64].

Thirdly, on nutritional considerations, Chauhan et al. [76] reported that ozone treatment caused significant loss of total phenolic compounds (expressed as gallic acid) in carrots sticks. However, in another study, carrots showed a conservation of many phenolic compounds: gallic acid, chlorogenic acid, caffeic acid, paracumaric acid, epicatechin, and catechin [63]. Evrendilek et al. [64] also reported a retention of ascorbic acid and total carotenoids with a decrease of the enzymatic activity of PPO (polyphenol oxidase) and POD (peroxidase). This decrease of enzymatic activity by ozone could be explained by the hyper-reactivity of ozone on the oxidative process and a protective mechanism to minimize the depletion of oxidizable substances [76].

A lot of studies focus on the immediate impact of the aqueous ozone treatment on carrot quality, and only a few studies have studied the effect during a short storage period under low temperatures [78] or at ambient temperature [74]. Paulikiene et al. [78] proposed a model to predict carrots’ storage duration. They concluded that carrots treated with ozonated water (1.9 mg L^−1^ at 3 °C) can be preserved 1.8 times longer than those washed only with tap water and 2.4 times longer than those in the control group without rinsing. The ozonized carrots sticks (10 mg L^−1^ during 10 min) kept under modified atmosphere packaging (MAP) for 30 days showed a maximum retention of overall sensory quality and microbial quality compared to those stored in air [76].

In conclusion, some physical and chemical changes of carrots can be controlled if the exposure time and concentration of ozone are kept as low as possible to inactivate microorganisms but still preserve their quality. Paulikiene et al. [78] worked on the creation of a cleaner production technology model for washing fresh carrots in order to reduce microbial contamination, increase resilience of the product, and reduce CO_2_ and SO_2_ emissions. It was established that ozone treatment washing technology and optimal storage conditions during storage could contribute to sustainable production, where production volumes are reduced, productivity is increased, less energy is used, a cleaner product is placed on the market, less waste is produced, and the product remains safe for a longer time by providing the right environment at the place of storage [78]. Moreover, with this ingenious technology model, water consumption was reduced by 96.16%, which is absolutely significant.

## 4. Lettuce and Salads

In the past twenty years, there has been an increasing demand for ready-to-eat vegetables, mainly because of their convenience and health benefits, and there is no sign of this demand slowing. Fresh cut salads, which are an important component of the human diet, have been one of the commodities with higher requests by supermarkets, restaurant like fast food services, and salad bars, and they represent more than 80% of the total production of fresh cut produce [81,82]. Lettuce provides a good source of minerals such as calcium and iron, vitamins A and C, and phytochemicals, including phenolic antioxidants, with considerable antioxidant potential [83].

The inner leaves of lettuce contain relatively low mesophilic bacteria, usually about 10^4^ CFU g^−1^, but counts in the packaged product are considerably higher due to the contamination involved by unit operations applied from the farm to the fork pathway (preparation, handling, cleaning, trimming, washing, drying, packaging, storage, and transport) and in particular, the shredder [84]. Postharvest is an important step to reduce contamination by foodborne pathogens on lettuces leaves, since these products are usually consumed raw. Disinfection represents one of the most critical processing steps influencing quality, safety, and shelf-life of salads. Moreover, leafy green vegetables are associated with 22% of the illnesses, and consumption of this fresh produce can be a risk factor for the transmission of known foodborne pathogens [85]. Lettuce is often contaminated by pathogenic bacteria such as *Salmonella*, *Listeria*, *Escherichia coli*, *Bacillus cereus*, *Campylobacter jejuni*, *Staphylococcus aureus*, and *Shigella* which could attach to open stomata, fissures in the cuticle or trichome, and leaf epidermal cell margin [55,86,87,88,89]. According to a study conducted by WHO, leafy green vegetables are the commodity group of highest concern as the cause of foodborne outbreaks [90]. Moreover, Garcia-Gimeno et al. [91] indicated that a maximum lactic acid bacterium count of 10^6^ CFU g^−1^ is an indicator of the beginning of spoilage of ready-to-eat salad. Their predictions of a product’s shelf-life indicated that a 4 °C storage could be as long as 8.7 days, which is longer than the 6 days established by manufacturers. This shelf-life is longer than current safety protocols on fresh-cut salad which define a shelf life of 5 to 7 days [92]. 

Chlorine is generally used in the fresh-cut industry to prevent the potential contamination and to extend the shelf-life of lettuce, but ozone treatment represents a sustainable technology that is able to improve the whole quality of lettuce and salads.

### 4.1. Effect of Continuous Ozone Exposure on Quality of Stored Lettuce

The first system used for ozone application as a way to increase the quality of stored lettuce is ozone in the gaseous phase because it is known that ozone molecules have longer half-life in air than in aqueous solution and higher diffusion rate. This application was investigated in a lot of different conditions such as hydroponically grown crops of butter head lettuce seeds and seedlings [93], in greenhouse growing of four-week-old lettuce [94], during a storage period immediately after harvesting [95] and for ready-to-eat leafy vegetables [55].

Ozone treatment of butter head lettuce seeds at 14 g h^−1^ during 30 min per day improves their germination, their uptake of elements, and their chemical composition and chlorophyll content, their physiological processes, their growth, and finally an increase their yield. A second ozone treatment of seedlings impairs the condition of plants and has a similar effect as tropospheric ozone in outside conditions with a decrease in yield [93]. Calatayud et al. [94] also observed that ozone treatment (8.2 to 83 nL L^−1^, 12 h day^−1^, 60 days) altered growth, decreased the mean weight and consequently productivity, and lowered its market value in two lettuce varieties studied. They observed significant differences between Morella and Valladolid varieties, the latter being more susceptible to ozone damage due to the lack of anthocyanins which have an antioxidant function [94].

Generally, treating food products with ozone gas can be achieved by adding low doses of ozone (from 0.1 to 10 µL L^−1^) to the storage atmosphere. When ozone is used as a gas immediately after harvest, the exposure time is longer than ozone dissolved in water–relatively long periods (from days to months). Galgano et al. [95] showed that the use of ozone at low concentrations (0.2 ppm) during 7 days at 4 °C did not alter sensory qualities such as the color stability of iceberg lettuce. Ozone was also able to inhibit the proliferation of total mesophilic bacteria and total coliforms, with a sporadic detection of yeasts and molds, *E. coli*, *Pseudomonas* [95]. These authors concluded that the use of ozone is effective in containing microbiological growth during chilling storage times of raw material, in comparison with just refrigeration. After processing in a similar manner, application of 1 µL L^−1^ on iceberg lettuce for 10 min did not alter color and showed a reduction in target microorganisms [55]. Other treatments involved high doses of ozone (from 100 to 10,000 µL L^−1^) for relatively short periods [87]. The objective of these treatments was to increase the shelf life of lettuces when refrigerated at 4 °C and it resulted in 1.0 to 1.5 log reductions in the numbers of both microorganisms but caused significant losses in important bioactive compounds: ascorbic acid, total phenolic contents, and antioxidant activity. In any case, it is important to point out that these gaseous ozone treatments are relatively seldom practiced in the food industry. Given the short shelf-life of green salads and lettuce, washing with aqueous ozone is favored.

### 4.2. Effect of Aqueous Ozone Exposure on the Quality of Lettuce

Ozone treatment of wash water is usually used in the sanitization treatment of heart of salad or raw salad and fresh cut lettuce. The objective of this treatment is to wash and decontaminate salads in order to market them immediately in refrigerated conditions, usually after a centrifugation and packaging them in a MAP or in the air. This sanitation is effective with different ozone application methods like bubbling, dipping, or immersion.

An assembly of research is presented in the following sections with emphasis on ozone’s impact on microbial inactivation and quality aspects of washing lettuce according to the type of treatment carried out: prewashing, immersion of salad leaves in a static ozonated water bath, and immersion of salad in a dynamic bath with ozone bubbling or with turbulence delivered by a pump (e.g., a process water recirculation pump).

#### 4.2.1. Prewashing Treatment

The precleaning treatment with ozonated water was tested on 80 heads of iceberg lettuce after a preparation step of removing and discarding wrapper leaves and excision of the core with knives and trimming [8,96]. This step was conducted by prewashing trimmed heads in ozonated water containing 1 mg L^−1^ ozone (120 s, 4 °C) at the beginning of the treatment, prior to shredding, and subsequent washing in tap water (4 °C, 90 s). The results obtained from the two published works are encouraging, as much on the microbiological aspects of the product as on the physical, chemical, and sensory qualities. The authors’ conclusions indicated that ozone treatment was effective in prolonging shelf-life of iceberg lettuce, but in order to achieve a disinfecting effect comparable with chlorine, a more efficient ozone application technology is required [8]. So, it is therefore with this objective in view that many authors have studied, on one hand, the effect of ozonated water on lettuce and salads in static conditions and on the other hand in dynamic conditions.

#### 4.2.2. Immersion in Ozonated Water without Continuous Ozone Injection

A majority of authors have studied the effectiveness of an ozonated water treatment in static ozonated water. This is a treatment that is easy to implement in a research laboratory and whose study contributes, as it were, to a preliminary research in order to study the feasibility of the tested treatment on a pilot or industrial scale. Table 6 shows the effect of this treatment on the overall quality of salads. 

Firstly, regarding the implementation of washing treatments by immersion of lettuce in static ozonated water, differences are observed according to the authors. Indeed, Akbas et al. [97] and other authors introduced ozone into water by means of an injection system until reaching the desired the ozone concentration. Some authors implemented an agitation system with magnetic stirring which may involve degassing the process water and therefore a more rapid decrease of ozone concentration [7,99] or no agitation and lettuce leaves simply float in stagnant water [63,97,98,100,101]. Usually, the ozonized solution was used immediately after the required concentration was reached, but it is most likely that the ozone concentration dropped during the treatment and no ozone measurements were taken during the dipping time or at the end of the immersion [7,63,97].

The large majority of authors analyzed the water quality over time and indicated that the water temperature is an important parameter to control. Increased temperatures lower the solubility of ozone and significantly influence its efficiency. Ozone activity is also influenced by the presence of organic compounds and pH variations. The turbidity of washing water below 5 NTU (Nephelometric Turbidity Unit) has no pronounced effect on inactivation with ozone [6]. Baur et al. [8] measured ozone concentrations in water, and they were stable within the 2 min prewashing procedure. However, when prewashing trimmed lettuce heads in ozonated water was practicable at chemical oxygen demand (COD) = 5.5 mg L^−1^, washing shredded lettuce in water containing 1 mg L^−1^ and 119 mg L^−1^ in COD was not feasible due to elevated ozone demand that resulted from the high organic load, which was mainly caused by release of cellular components through cutting.

Then, concerning visual appearance of lettuce, very few changes have been reported in the literature immediately after the washing procedure [97,99,100,102]. However, during storage, some changes were observed like a decrease of the overall quality and an increase of cut edge tissue browning when compared with chlorine treatment, which caused less browning on lettuce cut-edge [97,98]. Akbas et al. [97] indicated that changes in color parameters with time were in the form of an increase in a* values, indicating a loss of green pigment, a decrease in b* values, showing loss of yellowness, and a decrease in L* values, indicating darkening of iceberg lettuce. Wei et al. [100] showed that the lettuce browning increased greatly with the increase ozone concentration from 1 to 5 ppm and the storage time. When the ozone concentration was increased up to 10 mg L^−1^, the browning was increased up to about 6% and 9% after 2 and 3 weeks of storage. It was also indicated that browning was less affected by ozone treatment time. Total color changes, which indicated differences between initial and final color of lettuce, showed great changes between the end of the ozonated water treatment at 1 mg L^−1^ during 1 min and the end of the storage period of 10 days [99]. This variation in color is explained by an enzymatic browning causing the oxidation of the phenolic compounds over time; polyphenol oxidase (PPO) and peroxidase (POD) are involved in tissue discoloration. Moreover, when ozone treatment was compared to other sanitizing washing methods, lettuce treated with ozone gained significantly better scores than a sodium hypochlorite solution washing and an organic acids solution with citric and ascorbic acid washing [98] and high willingness to purchase scores rather than washing in a chlorine solution or in a combination with ozonated water and chlorine solution [7].

In spite of a decrease in the crispiness coefficient revealed by Rico et al. [99], no significant changes in texture and moisture were observed in lettuce samples dipped in ozonated water immediately after the treatment and even after a long storage in refrigeration conditions. This is in contrast with chlorine treatment which had an adverse impact on lettuce firmness [97,99,100,103]. Ozone treatment had a stronger inhibitory effect on the pectin methylesterase (PME) enzyme, which is responsible for cleaving the methoxyl groups from methylated pectic substances, generating free pectic acids [99]. PME activity decreased in lettuce upon ozone treatment, and a loss of firmness is observed. Firmness is an important quality attribute and may be decreased by loss of cell turgor due to water loss during storage. The moisture content of lettuce is also an important parameter for maintaining texture.

Secondly, concerning the microbial quality of salads, Wei et al. [100] showed that the inactivation of natural microflora increased with the ozone concentration. Dipping fresh-cut iceberg lettuce in ozonated water at 4 mg L^−1^ for 2 min (CT = 8 mg min^−1^ L^−1^) is efficient to reduce initial level of mesophilic, psychrotrophic bacteria, and *Enterobacteriaceae* by 1.3 to 1.7 log CFU g^−1^ [97]. Smaller reductions were observed at lower concentrations for aerobic mesophilic bacteria (0.46 and 0.67 log CFU g^−1^ in 15 and 30 min respectively) in fresh green leaf lettuce dipped in distilled water at an initial concentration of 0.5 ppm [63]. The resistance to ozone in descending order corresponded to mesophilic bacteria, psychrotrophic bacteria and yeasts, and finally, molds [100]. Yeasts and molds require an increasing ozone concentrations and longer exposure times for their inactivation by ozone treatment [63,100]. An ozone treatment on iceberg lettuce artificially contaminated by two pathogenic bacteria was conducted by Yuk et al. [101]. Ozone had no effect on the *L. monocytogenes* population of 6 log CFU g^−1^ even at the greatest treatment dose of 5 ppm and the longer treatment time of 5 min. However, this washing significantly reduced counts of *E. coli* up to 1.09 log UFC g^−1^ reduction in 5 min in ozonated water at 5 ppm and 22 °C without agitation. These authors concluded that the antimicrobial effect of ozone for killing bacteria on lettuce might be dependent on microbial species and strain, ozone concentration, contact time and temperature, and the delivery method (i.e., agitation, soaking, spraying, or bubbling). During 12 days of storage, microorganisms increased from 2 to 3 log CFU g^−1^ of aerobic mesophilic and psychrotrophic counts in lettuce [97,98]. Inhibition of microorganisms by ozone is due to oxidative changes in numerous cellular constituents, including proteins, unsaturated lipids, respiratory enzymes in cell membranes, peptidoglycans in cell envelopes, and nucleic acids in the cytoplasm [97].

The nutritional quality of lettuce was little studied. Despite the fact that the analysis methods of vitamin C and β-carotene are different, all the authors agreed that their concentrations remained constant immediately after the treatment [97,98] and during storage at 4 °C [97]. Moreover, ozone treatment has a beneficial effect on the microbial shelf life and quality of lettuce salads. As determined by appearance in the study of Garcia et al. [7], commercial lettuce salads treated with ozone had a shelf life of 21 days.

In conclusion, ozone treatment of lettuce by immersion in static ozonated water resulted in microbial reduction and conservation of physical appearance of products and avoided a loss in compounds of nutritional interest. Moreover, ozone, with its high oxidation potential, rapidly and efficiently oxides the organic matter that is suspended or dissolved in water [7]. It degrades dissolved pigments, like chlorophyll and carotenoids, from lettuce in the water and therefore keeps the COD, TOC (Total Organic Carbon) and turbidity of the water lower [7,98]. Moreover, dissolved ozone breaks down into oxygen without leaving any residue. Consequently, the quality of treatment water seems to remain constant for a longer period with ozone treatment (8 h), in comparison with other sanitizers as chlorine (4 h), making it available for longer reuse, and this helps to reduce water consumption, which is of considerable interest considering current concerns in terms of sustainable development [7].

Alexopoulos et al. [63] tested an experiment where ozone was supplied either at the beginning of the experiment, and the treatment was realized either in static water or continuously in dynamic conditions, and ozone concentration was kept constant at 0.5 ppm. Continuous ozone treatment was compared to ozone treatment conducted in static conditions, and significant differences were observed; continuous ozone treatment of wash water was more effective than immersion of vegetables in presaturated water. Getting a better ozone dispersion in the sanitizing solution is critical as findings published by various authors showed that sanitization treatments were more effective when ozone was bubbled rather than simply dipping vegetables in ozonated water [87]. This was explained by the film theory that states when ozone is bubbled into water, a liquid film forms at the ozone gas/water interface, and ozone becomes more concentrated in this liquid film than in the bulk liquid. Thus, higher microbial inactivation of *E. coli* was achieved in 2 min, when the contaminated lettuce was in contact with ozone bubbles (1.97 log UFC g^−1^ reduction), compared with dipping into the bulk liquid (1.17 log UFC g^−1^ reduction) [102].

#### 4.2.3. Immersion in Ozonated Water with Continuous Injection

In all studies presented in Table 7, ozone gas was delivered throughout the treatment duration to compensate for the loss of aqueous ozone due to quenching by organic matter, off gassing, and the short life of the ozone molecule. Organic matter consumes ozone and may compete with microorganisms, reducing the efficiency of ozone and hence requiring its continuous reintroduction into wash water.

Despite the fact that these aqueous treatments of lettuce leaves have been performed continuously and in dynamic conditions, few authors have measured the initial ozone concentration at the beginning of the treatment and throughout its duration. The majority of treatment facilities included manual sampling of ozonated water in order to measure residual ozone with commercial test kits from different suppliers [7,63,97], by direct measurement of UV absorption at 258 nm [104] or with the indigo method [87]. However, there is now a selective amperometric probe equipped with a flow cell and a temperature compensation sensor which are connected to a dissolved ozone analyzer and used to monitor dissolved ozone [86,103,106]. This online system is more efficient and faster than the kits available and indicates the ozone concentration in real time. Other facilities are able to be set to an objective concentration, and the concentration of ozonated water is monitored constantly using an integrated system [8,96,106].

Based on these analyses, Hassenberg et al. [86] demonstrated that an initial ozone concentration of 3.6 ppm resulting from the water ozone treatment process decreased to a very low concentration, ranging between 0.02 and 0.036 ppm, in the washer containing iceberg lettuce. On the contrary, Koseki et al. [104] indicated that the concentration in ozonated water with overflow is stable and does not decrease throughout treatment for 5 min at 3, 5, and 10 ppm. Similar results were demonstrated by Selma et al. [106] and Koseki et al. [104] after the same time.

In the studies presented by Garcia et al. [7] and Rosenblum et al. [105], the turbidity of treatment water remained constant for long periods with small salad quantities (100 and 300 g) and short ozone treatment of 10 [7] and 50 min [105]. On the contrary, Hassenberg et al. [86] indicated that in industrial conditions (450 kg of lettuce washing per hour), the high ozone consumption is due to the high reactivity of ozone with unsaturated organic compounds. Indeed, the TOC, TSS (total suspended solids), and COD content in the wash water rose continuously during the production time with further reductions from the addition of fresh water [86,105]. Addition of ozone to treatment water resulted in a noticeable delay in the increase in TOC content and a reduction in total TOC content, compared to a process without ozone or to another water treatment (with chlorine or citric acid).

Concerning the visual quality of lettuce, few studies have looked at this aspect, unlike studies on the impact on the microflora of the product. Treated lettuce conserves its sensory qualities (appearance, aroma, texture) after washing and during the storage period. Beltran et al. [103] observed that only the washing treatment with ozone (10–20 mg L^−1^ for 3–5 min) is effective in preventing tissue browning in shredded lettuce during storage at 4 °C in PP trays [103]. On the contrary, Koseki et al. [104] showed that ozonated water treatment causes rapid browning of lettuce during storage at 10 °C. This is explained by the authors by the strong oxidant activity of ozone: the lettuce tissue might have been damaged. Therefore, PAL activity was enhanced immediately after exposure, resulting in the initiation of browning. The extent of the browning also increased with increasing ozone concentrations.

Significant reductions of total bacteria (1.6 log CFU g^−1^) and coliforms (3 log CFU g^−1^) were measured in shredded iceberg lettuce washed in 10 and 20 mg L^−1^ ozonated water for 3 and 5 min [103]. Similar reductions were achieved for aerobic mesophilic bacteria, coliforms, yeasts, and molds at a continuous ozone treatment concentration with a system bubbling at 0.5 ppm after 15 min of treatment (CT = 7.5 mg min^−1^ L^−1^) and the reductions were even higher after 30 min of exposure, reaching 3.04 log CFU g^−1^ for aerobic mesophilic bacteria, 2.47 log CFU g^−1^ for coliforms, and 2.1 log CFU g^−1^ for yeasts and molds [63]. Similar log reductions were also achieved in the study of Gibson et al. [56] across all microorganism types inoculated on Romaine lettuce at significantly lower ozone concentrations (<1 mg L^−1^) and shorter contact times (2 min) compared to other studies [56,63]. However, the longer durations of treatment used in the study of Dev Kumar et al. [88] involved similar log reductions of *S. enterica*. Ozone treatment of Romaine lettuce artificially contaminated with *B. cereus* spores resulted in a reduction of spore concentration: 97.2% of spores were removed from the lettuce surface, with CT values ranging from 13.3 to 17.9 mg min^−1^ L^−1^. In contrast, the bactericidal effect of ozone increased uncorrelated to the increase of ozone concentration [104,106] and to prolonged treatment time [106]. However, *S. sonnei* inactivation was not increased when treatment time was prolonged, and counts remained unchanged from 3 up to 5 min treatment [106]. Compared with these studies, it is unclear why lower log reductions of bacteria on fresh lettuce are reported in some studies with relatively high ozone concentrations, while others reported reductions of more than 4 log CFU g^−1^. This may be explained by the arbitrary action of ozone towards bacteria and organic matter; this is especially critical if the treatment results in leaching of organic matter. These organic suspended solids may initially react with ozone by consuming ozone, as described by Cho et al. [39] and Hunt et al. [40], as opposed to bacteria that may be present in the washing water. The type of organic matter and the pH have a considerable effect on the percentage of disinfection by hydroxyl radicals. Inactivation of *Bacillus* endospores by ozone was approximately 20% more effective at pH 8.2 than at pH 5.6 and this inactivation was faster than in treatments with pH-controlled distilled water but slower than those with water containing humic acid [39]. Moreover, a small change in organic matter and long durations of treatment (more than 60 min) are not representative of washing practices used in the food industry [88]. Another explanation for these wide variations in the log reduction of microorganisms could be the acidification of the water caused by ozone. Indeed, a study by Hassenberg et al. [86] indicated that the pH of the wash water drops during the process by approximately 0.5 in 2 h of production in experiments with and without ozone. The reason for this decrease in pH is the leakage of cell content from damaged lettuce cells during washing. However, the ozone treatment obviously only marginally affects the pH value of the washing water. As described in Section 2.4.1 of this review, the optimal pH condition to maximize the antimicrobial action of ozone in washing water is acidic. At this pH, ozone is stable and its decomposition rate is slow.

Authors have indicated that the aerobic mesophilic total count level is similar in wash water treated with ozone during approximately 2 h of washing.

Moreover, it has been shown that bacterial growth in 6 days at 10 °C on lettuce treated with a sanitizer like ozone is more rapid (2.7 log CFU g^−1^) than that on lettuce washed with distilled water (1.7 log CFU g^−1^). This phenomenon is explained by the significant initial decrease in the bacterial population observed with ozone treatment, which reduces significantly the number of competing aerobic mesophilic bacteria (1.4 log CFU g^−1^ vs. 0.2 log CFU g^−1^ for washing with distilled water) and allows the remaining bacteria, that can be pathogenic, to thrive [104].

Therefore, ozone treatment has no effect on the nutritional quality of lettuce; washing with ozonated water results in better preservation of sugar content, compared to water-washed samples, and improved lettuce quality, as vitamin C concentration is preserved [86,104]. Similarly, treatment with ozonated water has been found to have no effect on chlorophyll a and b, ascorbic acid, total phenolic content, or antioxidant activity [87]. Active MAP was not effective in the preservation of vitamin C content, and a significant reduction of 75% was reached at the end of the storage period [103].

All of these studies show the interest in continuous dynamic treatment with ozonated water during storage as a way to increase the quality and the shelf life of the product, as shown in Table 8.

With ozone treatment, flume water is replaced daily, allowing for a flume water saving of at least 60% in comparison with chlorine treatment where the flume water quickly becomes discolored, laden with organic residues, and needs to be replaced every 2–3 h [107]. From an economic point of view, there are significant savings on the cost of fresh produce processing and wastewater treatment, on the gain of labor time resulting from less frequent changing of spent flume water, and of course savings of tap water from improving recycling practices. Furthermore, improving the quality of wastewater effluents enables the fresh food industry to meet the European effluent regulations that are set to become much strict as cleaner environments are more emphasized [108].

## 5. Tomatoes

The tomato is a very popular fruit cultivated in more than 100 countries, and world production during 2018 was 244 Mt [109]. The most important quality criteria for tomatoes are their red color, a firm but juicy texture, and a good flavor [110]. Tomatoes are consumed for their high nutritional qualities such as lycopene and ascorbic acid content and for their antioxidant, anti-inflammatory, and anticancer activity [111]. However, tomatoes can be contaminated with foodborne pathogens such as *Salmonella* or *Norovirus* [112] and can be degraded during storage by microorganisms and particularly by fungi. Usually harvested by hand into boxes, tomatoes are transported to packing houses where fresh fruits are minimally processed before storage. Tomatoes are not submitted to physical treatments that will eliminate the presence of *Salmonella* and *Norovirus* [112]. To avoid these contaminants, good agricultural and good manufacturing practices are required. The two main factors for controlling microorganism development are wash water quality and storage conditions. Ozone can be applied during washing or during storage.

### 5.1. Effect of Exposure to Continuous Gaseous Ozone on the Quality of Stored Tomatoes

Storage of tomatoes is an important step for ripening and for extending their shelf life. Due to their high concentrations of water and nutrients, tomatoes are very vulnerable to microbial degradation, particularly by yeasts and molds. Moreover, the shelf life of tomatoes is linked to different abiotic stresses experienced during the ripening and harvesting phases [113]. Storage in a positive-pressure chamber is generally used for preserving tomatoes but this influences flesh firmness and the organoleptic and functional properties of these fruits [113]. Moreover, the gas exchange (e.g., CO_2_, O_2_, ethylene) occurring during ripening and storage results in the liberation of water that can induce microbial development. This water can lead to internalization of various hazardous bacteria and fungi and particularly more on stem scar tissue than smooth tissue [114]. To avoid these developments, gaseous ozone treatment can be used before or during storage and particularly when refrigeration is not possible.

Several authors have studied the effectiveness of ozone for decontaminating tomatoes (pathogens or fungi) while retaining the nutritional and organoleptic qualities of different types of red ripe tomatoes, on green tomatoes for ripening, or on packaged tomatoes (Table 9).

For ripe tomatoes, different varieties have been tested: cherry, beefsteak, and grape tomatoes. Cherry tomatoes were inoculated with *Salmonella* *enteritidis* onto the surface at low and high doses (3 and 7 log CFU tomato^−1^) [115] and stored for 1 or 4 h to encourage bacterial attachment. For the low inoculum, no bacteria were detected after 1 and 4 h of storage if tomatoes were treated with 10 mg L^−1^ for 5 and 10 min, respectively. For the high inoculum, complete bacterial destruction was achieved after 15 min at 20 mg L^−1^ for 4 h attachment time. To reduce the time, a higher concentration, 30 mg L^−1^, was used but that led to a red to yellow change. Bridges et al. [70] studied the effectiveness of gaseous ozone for destroying a cocktail of pathogens (*Listeria monocytogenes*, *Escherichia coli* (STEC), *S. enterica*) at a final contamination above 6.5 log CFU g^−1^ on beefsteak tomatoes. After 5 h of exposure at 1.71 µg O_3_ g^−1^ produce, a maximal reduction of 1.6 log CFU g^−1^ was observed for *E. coli* STEC, and for the two other genera, *Salmonella* and *Listeria*, a reduction of 1.1 log CFU g^−1^ was observed. However, while the higher concentration and longer duration of treatment permitted greater bacterial destruction, unfortunately, a bleaching of the tomato epidermidis was noted. Grape tomatoes inoculated on their smooth surface and scar stem with *Salmonella* or uninoculated were treated with different concentrations of ozone (1.71, 3.43, and 6.85 mg L^−1^) for 2 or 4 h [116]. Whatever the tissue (stem scar or smooth zone), a concentration of 6.85 mg L^−1^ for 2 h was required to observe a 2 log CFU fruit^−1^ reduction of *Salmonella*. Considering native microbiota, regularly evaluated during storage at 10 °C, a reduction of total plate count was observed at days 1 and 7 of storage for concentrations of 3.43 and 6.85 mg L^−1^, but yeast and mold populations were not affected whatever the gaseous ozone treatment. Moreover, visual degradation and off-notes aroma were noted during these experiments. After exposure to a concentration of 6.85 mg L^−1^ for 4 h, the tomatoes appeared wet, suggesting rupture of the skin. Two nutritional markers were followed during storage. At day 1, no impact on ascorbic acid and lycopene content was detected. However, during storage, a progressive decrease of these compounds was detected until finally only 1/3 of the ascorbic acid remained at day 21. Lycopene degradation was correlated with red color alteration. To improve their results, the authors carried out new experiments using 800 and 1600 ppm for 30 min, coupled or not coupled with hydrogen peroxide, after inoculation of *Salmonella* on the smooth surface and scar stem [117]. Only a 0.5 log CFU fruit^−1^ reduction was obtained when ozone gas was used alone, but addition of aerosolized hydrogen peroxide to the ozone gas treatment induced a 5.2 log CFU fruit^−1^ reduction on the smooth surface.

Tzortzakis et al. [118] focused on the impact of ozone on the destruction of fungi and the nutritional and sensory qualities of fully ripe tomatoes inoculated with *Botrytis cinerea* and exposed to charcoal-filtered clean air or low-level ozone enrichment (0.1 μmol mol^−1^) at 13 °C. Ozone enrichment resulted in a substantial decline in spore production/viability of *B. cinerea* and the development of visible lesions in all treated fruit. Considering the quality of tomatoes, whatever the conditions tested (range from 0.005 to 1.0 μmol mol^−1^ ozone, at 13 °C and 95% relative humidity), there was no impact on weight loss, antioxidant status, CO_2_/H_2_O exchange, or the content of organic acids, total phenol, or vitamin C [119]. However, sensory analysis revealed that tomatoes treated with 0.15 µmol mol^−1^ ozone were appreciated more than those treated under other conditions. The authors suggested that pesticides used to avoid fungal development during storage could be replaced by ozone.

Sometimes tomatoes are harvested mature-green and the ripening is carried out at room conditions where temperature and humidity are uncontrolled. These conditions lead to fungal development and using a disinfectant such as ozone could help preserve quality. Zambre et al. [120] evaluated ripening under ozone treatment at different temperatures with two indices: red color development and the rotting index based on 75% of maximum spoilage. Tomatoes at different stages of ripening were put in an ozone chamber and treated for 10 min at 20, 35, and 50 ppm, packaged individually in unsealed bags and kept at 15, 20, and 35 °C at 68 ± 3% relative humidity. The best result was achieved when the tomatoes were treated at 35 ppm for 10 min and stored at 15 °C, which led to a 10-day extension of shelf life with ripening delayed by about 3.6 days. However, an increase of storage temperature annihilated the benefits of ozone treatment. Ozone treatment induced a reduction of initial microbial count, whereas lower temperature reduced the rate of microbial growth; consequently, both induced a longer shelf life. Venta et al. [121] evaluated the impact of gaseous ozone on some physical-chemical parameters and loss during the postharvest period in unripe tomatoes in Cuba. Exposure of green tomatoes to 25 mg m^−3^ ozone for 2 h day^−1^ for 16 days gave the best results in terms of firmness, loss of weight, and spoilage, but in this case, the tomatoes had a lower lycopene and ascorbic acid content than the control. Ozone treatment extended the shelf life of tomatoes (only 14% damaged fruit versus 54% for the control), probably by decreasing the rate of ripening. However, if a high concentration is used, some damage linked to ozone treatment is detected. Optimal conditions must be found for improving tomato quality.

The fact that ozone modifies the ripening process has already been described previously [123], and the changes of physiological state can be explained with proteomics analysis. Tzortzakis et al. [123] compared the protein profiles of tomatoes stored for 1 week under four conditions: ozone (0.05 µmol L^−1^), wound inoculation with *B. cinerea* (a 2.5 mm mycelial plug in a wound), and with or without treatment (charcoal-filtered clean air) after two pretreatments, 1 week under ozone (0.05 µmol L^−1^) or 1 week under clean air. These pretreatments were carried out to evaluate a potential memory effect linked to the ozone treatment. Ozone treatment induced a higher content of proteins, but this change was reversible when tomatoes were removed from the ozone-enriched atmosphere. Tomato proteomes were clearly modified when fruits were subjected to ozone and/or *B. cinerea* wound inoculation, but proteome shifts were qualitatively suppressed when tomatoes under ozone were placed in clean air or when tomatoes inoculated with *B. cinerea* were put under an ozone atmosphere. This last observation was explained by the fact that in the presence of ozone, oxidative stress proteins are synthetized and that prepares the tomatoes to respond to pathogens. Moreover, ozone treatment downregulates some proteins implicated in ethylene production. Consequently, a lower rate of ethylene would slow the development of pathogens but also reduce ripening.

Tomatoes can be treated using an in-package ozone treatment system [122]. Tomatoes inoculated with *L. innocua*, *Salmonella* Typhimurium, or *E. coli* O157:H7 were put into a sealed bag and placed in a treatment chamber for generating ozone directly into the bag. A reduction of between 1.8 and 6 log CFU unit^−1^ was observed depending on the strain and the area considered (surface or scar stem). Firmness and color of tomatoes stored for 22 days at 22 °C were not noticeably affected by the ozone treatment step in the package.

As some works have demonstrated, ozone can be applied for tomato disinfection and storage, with benefits such as extending the shelf life and reducing the microbial population. However, the maturation stages, environmental conditions such as temperature and humidity, and the variety of tomatoes (weight of product) must be considered if we want to keep sensory and nutritional qualities. Ozone gas can be used to manage the ripening phase. This could be very interesting for wholesalers who could thus delay the ripening phase and put tomatoes on the market when necessary.

### 5.2. Effect of Exposure to Aqueous Ozone on the Quality of Tomatoes

Because of the fragility of the tomato fruit, few authors have studied the treatment of tomatoes in ozonated water.

The impact of the treatment on the sanitary quality of the product has been particularly studied. Venta et al. [121] evaluated the application of ozone in gaseous and aqueous phases for postharvest disinfection of tomatoes. The application of aqueous ozone using a dissolved ozone concentration of between 0.5 and 1 mg L^−1^ during a 15, 22, or 30 min immersion at 27 °C and pH = 7.2 achieved significant microbial reduction of *E. coli*. Moreover, Chaidez et al. [124] analyzed the impact of immersion or spraying on fresh ripe tomatoes surface-inoculated with *S.* Typhimurium. Contact times of about 30 and 120 s with 1 and 2 mg L^−1^ ozonated water at 25 °C and pH = 7.0 were efficient for reducing *S.* Typhimurium. However, it was demonstrated that the use of ozonated water both in immersion and spraying applications is suggested when water turbidity remains low. Xu and Wu [125] confirmed that treatment with ozonated water for 1, 5, or 10 min at room temperature and pH = 5.6 is efficient for the inactivation of *S. enterica*, with a concentration of dissolved ozone in the water of 6.25 ppm. The authors also assumed that this large reduction is due to the smooth surface of tomatoes which may allow more direct contact between ozone and bacteria. According to Mustapha et al. [126], aqueous ozone is not very efficient for washing cherry tomatoes as they observed a very small reduction of mesophilic bacteria, yeasts, and molds (<1 log CFU g^−1^). Treatment of tomato slices with 0.4 mg L^−1^ ozonated water for 3 min achieved a significant reduction of mesophilic and psychrotrophic bacteria, and the yeast load.

However, it is deplorable that few studies have focused on maintaining the shelf life of tomatoes and their nutritional and sensorial qualities. Mustapha et al. [126] washed fully mature red round-like cherry tomatoes in ozonated water at a concentration of 0.85 mg L^−1^ for 5, 10 or 15 min and observed no detrimental effect on the physicochemical properties (i.e., firmness, maturity index, total soluble solids content, titratable acidity, pH, electrolyte leakage), bioactive compounds, or antioxidants during 21 days of refrigerated storage [126]. Treatment with 0.4 mg L^−1^ ozone for 3 min achieved the best firmness retention of tomato slices and did not affect the total acidity, pH, total soluble solids, organic acids, or sensorial quality [127].

Finally, Xu and Wu [125] and Mustapha et al. [126] suggested that a combined system of ozonated water washing and mild heat at 50 °C or mono-mode and dual-mode frequency irradiation results in greater microbial reduction without a detrimental effect on the tomatoes, except on the firmness.

In conclusion, spraying or immersion in ozonated water seems to be efficient for maintaining the sensory, nutritional, and physicochemical properties of tomatoes. Moreover, Rodrigues et al. [128] reported that concentrated ozonated water at 3 mg L^−1^ is effective in removing pesticide residues in tomatoes, reaching a reduction of 70% to 90% of the residues.

## 6. Synthesis and Conclusions

**Figure 3:**
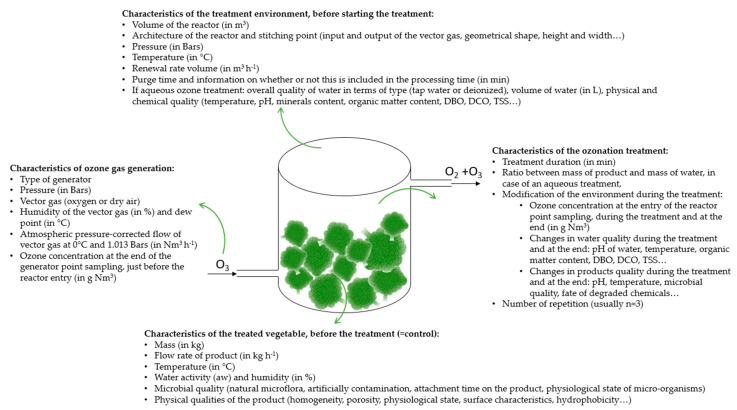
Synthesis of ozone treatment of vegetables with essential characteristics that must be indicated to characterize the ozone treatment.

At the end of this review, it becomes very clear that the use of ozone, in gaseous or water-solubilized form, may be used for the preservation of fresh vegetables (i.e., control of the growth of spoilage and pathogenic microorganisms as well as the preservation of quality characteristics) because this molecule is endowed with antimicrobial activity, thanks to its oxidative capacity against proteins, lipids, enzymes, nucleic acids, membranes, and other cellular constituents. However, as we have been able to see during this review, the performance which can be reached during the use of ozone is very strongly dependent on the general conditions of this implementation. Most of these considerations are synthesized in Figure 3.

In most cases, the proper consideration and management of the major parameters (Table 2) allows users to find a real compromise between the effectiveness of controlling microorganism growth and preservation of the nutritional and organoleptic properties of the products. However, at the same time, some researchers/potential users believe that this technology is influenced by too many factors and so is not promising from an industrial point of view with regard to its strict performance (i.e., a reduction in the microbial load of 1–2 log as reported in most of the papers consulted).

Today, in order to overcome these criticisms, which mainly relate to the limited effectiveness (i.e., limitation of the inactivation of microorganisms) of ozone under applied conditions, new technological approaches may be considered. These approaches are based on the concept of “hurdle technology”. The hurdle concept (generally known as combined methods, combination preservation, combined processes, barrier technology, or combination techniques) has become a promising technological approach that can simultaneously reduce losses of nutritional and sensory quality and improve food safety [129,130]. In fact, hurdle technology shows synergistic effects while using various mechanisms for the inhibition or inactivation of targeted microorganisms [131,132]. Some successful combinations of techniques using ozone have been already reported (i.e., ozone and ultraviolet-C [133], ozone and an advanced oxidative process [134,135], ozone and mild heat at 50 °C or mono-mode and dual-mode frequency irradiation [125,126], ozone pretreatment combined with modified atmosphere packaging [136,137,138], ozone treatment in combination with passive refrigeration [139], and vacuum cooling [140]). Most of these approaches combining ozone with another technique are promising because they can potentially enhance the antimicrobial effects, reduce the severity of treatment required to obtain a given level of microbial inactivation, and prevent the proliferation of microorganisms surviving after ozone treatment. In view of the results obtained, it therefore seems interesting to continue and expand the experiments being carried out in this field.

## Figures and Tables

**Table 1:** Main physicochemical properties of ozone [23].

Property	Value
Molecular formula	O_3_
Cas Number	10028-15-6
Molecular Weight	47.998 g mol^−1^
Melting Temperature (1 atm.)	−192.5 ± 0.4 °C
Boiling Temperature (1 atm.)	−111.9 ± 0.3 °C
Critical Temperature	−12.1 °C
Critical Pressure	54.6 atm.
Density (0 °C, 1 atm.)	2.14 g L^−1^
Diffusivity (20 °C)	1.79 × 10^−9^ m^2^ s^−1^ (liquid form)/1.46 × 10^−5^ m^2^ s^−1^ (gaseous form)
Oxidation Potential	2.07 V

**Table 2:** Extrinsic and intrinsic factors influencing efficacy of ozone.

	Parameters	Factors
Extrinsic factors	Water quality	pH, organic matter, pressure, and temperature
	Air quality	Air relative humidity
	Ozone treatment	Concentration and treatment time application method
Intrinsic factors	Food product	Type of fruit and vegetable, weight, characteristics of the product surface, and surface area. Activity of water (aw)
	Microbial load	Characteristics of microbial strains, physical state of bacterial strains, natural microflora, artificially inoculated microorganisms, and population size

**Table 3:** Overview of the impact of continuous gaseous ozone treatment on quality and safety characteristics of stored carrots.

Ozone Generation	Treatment Conditions	Produce	Microbial Characteristics	Quality Characteristics	Author’s Conclusions	References
Tri-Ox, Swindon. O_3_ production: air, 76.5 µL L^−1^, flow rates: 0 to 0.4 L min^−1^	0, 7.5, 15 and 60 µL L^−1^, 0.5 L min^−1^ total flow, 2–16 °C, 8 h daily for 28 days	Fresh carrots artificially contaminated with *S. sclerotiorum* and *B. cinerea*	50% reduction of daily growth rate at 60 µL L^−1^	Lighter carrots with less intense color, physiological damage (dry white blotches, brown water-soaked lesions on leaves), increase of respiration rate with increase of ozone concentration	Optimum treatment conditions: 15 µL L^−1^ for 8 h at 2 °C	[68]
Aqua air ozone generator SF300, Simpson environmental Corp.	450 or 600 ppb, 5 or 20 °C, 97% RH, 48 h	Fresh carrots artificially contaminated with *S. sclerotiorum* and *B. cinerea*	53.2% reduction of daily growth rate at 450 ppb, reduced lesions size and height of the aerial mycelium	No significant effect on color during 12 storage days	Optimum treatment conditions: 450 ppb for 48 h	[69]
Aqua air ozone generator SF300, Simpson environmental Corp.	300 or 1000 nL L^−1^, 10 °C, 0 to 4 days	Fresh carrots artificially contaminated with *S. sclerotiorum* and *B. cinerea*	Larger effect on inducing resistance in carrots to *B. cinerea* compared with *S. sclerotiorum*	Reduction of firmness, increase of respiration rate with production of stress volatiles, ethanol and hexanal, and decrease of sucrose concentration	Limited effects of tested ozone treatment	[70]
Aqua air ozone generator SF300, Simpson environmental Corp.	50 nL L^−1^, 0.5 °C, >95% RH, 6 months	Fresh carrots artificially contaminated with *S. sclerotiorum* and *B. cinerea*	Reduction of lesion size and rate of expansion	No effect on fresh weight loss, sprouting of carrot crowns, concentration. Increase of isocoumarin and brown discoloration of periderm	Application of much lower concentration as 50 nL L^−1^	[71]
Clear water Tech, Inc. O_3_ production: oxygen, flow rate: 1 L min^−1^	2.1, 5.2 and 7.6 mg L^−1^, 22 °C, 80% RH, 5, 10, or 15 min	Baby carrots inoculated with *E. coli* (7.8 log CFU g^−1^)	Lethal effect toward *E. coli* by 1.11–2.64 log CFU g^−1^	No decolorization	Increase of bactericidal effect with concentration and length of exposure	[72]
LG-7 generator, Del-Ozone. O_3_ production: oxygen, flow rate: 2 L min^−1^	428 or 856 mg m^−3^, 2.5 or 5 h	Baby-cut carrots inoculated with strains of *E. coli*, *Listeria* and *Salmonella*	Reduction of 1.2 log CFU g^−1^ of *E. coli*, 0.8 of *Listeria* and 0.5 of *Salmonella*	Noticeable bleaching	Increase of bactericidal effect with concentration and exposure time	[73]
O&L3.ORM, Ozone & Life. O_3_ production: oxygen, flow rate: 2 L min^−1^	1–5 mg L^−1^, 3.9–24.1 °C, 9.5–110.5 min	Fresh carrots	Not determined	After the treatment: no modification of L*, a*, b*, weight, firmness, pH and soluble solids (SS) and after 5 days storage: no modification of L*, a*, b*, weight, firmness, pH and increase of SS	Increase the shelf-life of carrots	[74]

**Table 4:** Synthetized results of the impact of ozone treatment on quality and safety characteristics of stored carrots, listed in ascending order of CT. A “+” indicates a retention or an improvement of the quality and a “−” indicates a noticeably negative change.

Reference	Maximal Applied CT ^1^ (mg min^−1^ L^−1^)	Maximal Tested Processing Rate (mg kg^−1^)	Visual Quality	Microbial Quality	Physical Quality	Nutritional Quality
[69]	1.73	/	+	+		
[70]	5.76	/		+	−	−
[71]	12.96	/	−	+	+	
[72]	114	1.71	+	+		
[73]	256.8	/	+	+		
[74]	552.5	/	+		+	
[68]	804.6	/	−	+		

^1^ Calculated from available information in the cited literature.

**Table 5:** Overview of the impact of an ozone washing treatment on quality and safety characteristics of washing fresh carrots.

Ozone Generation	Treatment Conditions	Applied CT (mg min^−1^ L^−1^)	Produce	Microbial Characteristics	Quality Characteristics	Authors Conclusions	References
O & L3.ORM, Ozone & Life. O_3_ production: oxygen, flow rate: 2 L min^−1^	2–10 mg L^−1^, 3.9–24.1 °C, 9.5–110.5 min	Between 19 and 1105	Fresh carrots	Not determined	After the treatment: no modification of L*, a*, b*, weight, firmness, and soluble solids (SS) but a decrease of pH. After 5 days storage: no modification of L*, a*, b*, weight, firmness, pH and increase of SS	Minor modifications of carrot quality with ozone dissolved in water after the treatment and during a storage for 5 days (18 °C, 80% RH)	[74]
O_3_ generator, Yeojen	8.2 g m^−3^, 5 and 15 min	41 and 125	Fresh carrots	Complete inactivation of 4.8 log CFU g^−1^ *E. coli* O157:H7. Significant reduction in total mesophilic aerobic, yeasts and molds, coliform bacteria, and *S. enteridis*.	No significant change in physical properties: Brix degree, titratable acidity, conductivity, browning index, and firmness. No significant change in chemical properties: ascorbic acid concentration, phenolic compounds, and carotenes. Decrease of organic acid content	8.2 g m^−3^ during 5 min is the best nonthermal treatment to maintain carrots quality and safety	[64]
Not determined	Spraying ozonated water at 1.9 mg L^−1^ for 2 min	3.8	Fresh carrots, two months after their harvest	Significant decrease of molds after the treatment (2.5 log CFU mL^−1^ reduction) and smaller concentration after 28 d storage at 3 °C (3.2 log CFU mL^−1^)	Linear constant and consistent decrease of carrot weight during 36 d storage	Carrots treated with ozonated water can be preserved 1.8 times longer than those washed with tap water	[78]
Clear water Tech, Inc. O_3_ production: oxygen, flow rate: 1 L min^−1^	5.2, 9.7 and 16.5 mg L^−1^, 22 °C, 120 rpm, 1, 5, 10 or 15 min	Between 5.2 and 247.5	Baby carrots inoculated with *E. coli* at 7.82 log CFU g^−1^	Significant lethal effect toward *E. coli* by a maximum of 1.85 log CFU g^−1^ at 16.5 mg L^−1^ for 15 min	No decolorization	Increase of bactericidal effect with concentration (>9.7 mg L^−1^) and length of exposure (>10 min)	[72]
Model VK-800A, Vege Kleen. O_3_ production: oxygen, 200 mg h^−1^	10 mg L^−1^, 5–7 °C, 10 min	100	Carrot sticks stored in air or modified atmosphere packaging (MAP)	Reduction of total plate count by 1 to 2 log CFU g^−1^	Reduction in total phenolics, PPO and POD activities, respiration and ethylene rate, retention of acid ascorbic, total carotenoids and lesser color changes	Lesser increase in microbial count and maximum quality and sensory score with association of ozone treatment and MAP during 30 d storage	[76]
Model Lab 11, Pacific ozone. O_3_ production: air, 3.4 V, 6 psi, 2 L min^−1^	5 ppm, 20 °C, 3–15 min	Between 15 and 75	Carrots in small discs contaminated with *E. coli*	Low degree of inactivation even after 15 min	Changes in color after processing: increase of luminosity L*, loss of redness-greenness a* and b*, reduction of chroma C*, and significant white discoloration		[62]
OZ5 generator, SPO3. O_3_ production: oxygen, 5 g h^−1^	1 ppm, 5 °C, 5min	5	Peeled carrots and shredded carrots	Microbial reduction up to 0.4 log CFU g^−1^ total mesophilic aerobic count and 0.6–0.7 log CFU g^−1^ yeasts and molds	Decrease of soluble solid content, color changes. No pH modification	Minimal quality changes for peeled carrots compared to shredded carrots	[79]
SOZ-YMS ozone generator. O_3_ production: oxygen	1, 2 and 3 mg L^−1^, 20 °C, 60, 120 and 180 s	Between 1 and 9	Shredded carrots	Significant decrease in total plate count (TPC) of 1.2 log CFU g^−1^ in 180 sec at 2 and 3 mg L^−1^. Significant reduction of yeasts of 1.4 log CFU g^−1^	Not determined	Better microbiological safety with increase of concentration and length of exposure	[80]

**Table 6:** Overview of the impact of ozonated water washing treatment on quality and safety characteristics of salads after washing and during conservation at 4 °C.

Ozone Generation	Treatment Conditions	Produce	Conservation	Microbial Quality after Washing	Physical, Chemical and Nutritional Qualities after Washing	Qualities after Conservation	References
Mikron Makina Ktd generator, O_2_	1.5 L of distilled water at 20 °C, pH = 7.8, 4 mg L^−1^, 2 min	75 g iceberg lettuce cut into 5 by 2 cm strips	12 days in 150 g plastic bag (PP) at 4 °C	Reduction of 1.7 log CFU g^−1^ of mesophilic bacteria, 1.5 log CFUg^−1^ of psychrotrophic bacteria and 1.3 log CFU g^−1^ *Enterobacteriaceae*	Conservation of color, texture, and moisture. No significant change in vitamin C and β-carotene content	Increase of 3 log CFU g^−1^ of all studied microorganisms after 12 d storage. Conservation of texture and moisture. Decrease of L* and b* and increase of a*. Decrease in vitamin C and β-carotene content	[97]
Air&Water System PC1325, air	5 L of distilled water at 15–17 °C, pH = 6.5 to 7.3, 0.5 mg L^−1^, 5 to 30 min, turbidity 2.7 NTU	200 g fresh green leaf lettuce	/	Reduction of 0.46/3.27 log CFU g^−1^ for aerobic mesophilic bacteria	/	/	[63]
Active Oxygen Generator, Golden Buffalo, 4L min^−1^, 215 Pa	1 L of distilled water at 4 °C, 2.5, 5 or 7.5 mg L^−1^, with stirring, 10 min	100 g of iceberg lettuce cut into 2 by 3 cm strips	25 days at 4 °C	Reduction of 0.6–0.8 log CFU g^−1^ of aerobic counts and 0.5–0.7 log CFU g^−1^ of psychrotrophic whatever the concentration between 2.5 and 7.5 mg L^−1^	High willingness to purchase score after treatment	High willingness to purchase score during storage. More slowly degradation. Acceptable shelf life of 21 days	[7]
Mini Ozone injection system, Ozone solution, oxygen, 30 g h^−1^	5 L of distilled water at 10 °C, 2 ppm, 2 min (optimum condition)	250 g of shredded green leaf lettuce	12 days at 4 °C	Reduction by about 1.5, 1.1 and 1.5 log CFU g^−1^ for aerobic mesophilic count, psychrotrophic count, and *Enterobacteriaceae*, 2 log CFU g^−1^ reduction of *L. monocytogenes*	High overall quality (9/10), no cut edge tissue browning, acceptable firmness and aroma. No significant change in vitamin C and β-carotene	Increase of 2 and 3 log CFU g^−1^ of aerobic mesophilic and psychrotrophic counts, suppression of the growth of *Enterobacteriaceae.* Good quality until day 7 (8/10), decrease of overall quality at day 12 (3.1/10) and better scores in all sensory parameters, in comparison with other treatments. No significant change in vitamin C, significant loss (35%) of β-carotene	[98]
Oxygen generator, model HV-103, 2.5 L min^−1^	Distilled water at 4 °C, 1 mg L^−1^, 1 min with agitation	200 g of fresh cut iceberg lettuce	10 days at 4 °C in PP bags	/	Good sensory evaluation of fresh appearance	Good sensory evaluation of fresh appearance, decrease of crispiness. Reduction of PPO and PME activity and increase of POD activity	[99]
Lab2B generator Ozonia	Milli-Q-water, at 4 °C, pH = 6 or 7, 3–10 min, 1, 3, 6 and 10 mg L^−1^	Shredded lettuce samples cut into 3.5 by 3.5 cm	21 days at 4 °C	Reduction of 0.74, 1.17, and 0.99 log CFU g^−1^ of mesophilic, psychrotrophic and yeasts and molds after ozone treatment at 10 mg L^−1^	Little decrease in lettuce firmness when increasing ozone concentration, no typical browning appearance	Little change in lettuce firmness throughout 21 days of storage, increase of typical browning	[100]
Green water ozone generator GW-1000	Water at 22 °C, 0.5 to 5 min at 1, 3 and 5 ppm, without agitation	Iceberg lettuce cut into 3 by 3 cm contaminated by *E. coli* and *L. monocytogenes*	/	No effect on *L. monocytogenes* population. Significant reduction of *E. coli* at 3 and 5 ppm up to 1.09 log CFU g^−1^ reduction with 5 ppm for 5 min	/	Increase of survivors of *E. coli* and *L. monocytogenes*	[101]

**Table 7:** Overview of the impact of continuously and dynamic washing treatment with ozonated water on quality and safety characteristics of salads after washing and during conservation.

Ozone Generation	Treatment Conditions	Produce	Conservation	Microbial Quality after Washing	Physical, Chemical and Nutritional Qualities after Washing	Qualities after Conservation	References
Air&Water System PC1325, O_2_	5 L of distilled water at 15–17 °C, pH = 6.5 to 7.3, 0.5 mg L^−1^ (continuously), 5 to 30 min, turbidity 2.7 NTU	200 g fresh green leaf lettuce	/	Reduction of 1.7/3.04 log CFU g^−1^ for aerobic mesophilic bacteria, 2.2/2.47 log CFU g^−1^ for coliforms and 2/2.1 log CFU g^−1^ for yeasts and molds in 15/30 min with continuous exposure	/	/	[63]
Generator model 1A steriline, 3 g h^−1^, 0.012 mm^3^ h^−1^, closing circuit	50 L deionized water at 4 or 8 °C, pH = 7.5, 10 and 20 mg L^−1^, 3 to 5 min	2 kg shredded iceberg lettuce	13 days at 4 °C, in PP trays in 2 different atmospheres	Reduction of 1.6 log CFU g^−1^ of total microorganisms and 3 log CFU g^−1^ of coliforms	Conservation of sensory quality (no promoting of browning, excellent visual quality, full aroma) and texture. Lower content of vitamin C and polyphenol	Slow microbial growth throughout 13 days of storage (1.8 log CFU g^−1^). No significant difference in the visual appearance, no affection of texture and conservation of full aroma. Conservation of vitamin C content and variation of polyphenol concentration similar to the control	[103]
BWOSS (Batch Wash Ozone Sanitation System)	34.1 L of water at 4 to 26 °C, <1 mg L^−1^, 2, 15 or 30 min, organic load	3 to 4 external leaves of seven hearts of romaine lettuce artificially contaminated	/	Reduction of 2.7 log CFU g^−1^ of *E. coli* and 2.9 log CFU g^−1^ of *S. thyphimurium* and *L. innocula* in 2 min. Reduction > 3 log CFU g^−1^ in 15 min and >4 log CFU g^−1^ in 30 min	/	/	[56]
Forever Ozone OG-5- G-BB	2 L of PBS at 1–4 °C, 0.17–0.23 mg L^−1^, 60, 90 and 120 min	10 g contaminated iceberg lettuce leaves with *S. enterica*	/	Decrease of 1.76, 1.67 and 2.09 log CFU g^−1^ in 60, 90 and 120 min	/	/	[88]
Coolzon 8, BMT Wassertechnik, 7.2 g h^−1^, 2m^3^ h^−1^, 3.6 pp m	2 m^3^ of tap water at 4–6 °C, 0.02 to 0.036 ppm	450 kg h^−1^ of iceberg lettuce shredded into 3 by 3 cm pieces	6 days of storage at 4 °C	10^5^ CFU g^−1^ of aerobic mesophilic total count and no detection of *E. coli* and *Salmonella*	Increase of vitamin C content by about 8% and total sugar content by 12%	Increase by 2 to 2.5 log units to 10^7^ CFU g^−1^. Decrease of vitamin C and total sugar content respectively by about 10% and 14%	[86]
OG20 Opal, oxygen feed gas, 20 g h^−1^, 827 mL min^−1^	1 L of distilled water at 5 °C, 15 min, 12 mg L^−1^	10 g of lettuce uniform in size and color	/	2 log CFU g^−1^ reduction in *E. coli* and *L. innocula*	No detrimental effect on chlorophyll a and b, ascorbic acid, total phenolic content, and antioxidant activity	/	[87]
Flow type electrolytic ozone generator Do-30, Kobe Steel, 3 L min^−1^,	5 L of water at ambient temperature, 5 min, 3, 5 and 10 ppm	350 g of iceberg lettuce cut into of 5 by 5 cm pieces	6 days at 10 °C in plastic PE film	Decline of aerobic mesophilic bacteria of 1 log CFU g^−1^ at 3 ppm. No further reduction above 5ppm ozone log CFU g^−1^	Increase of a* value indicating rapid onset browning. Increase of PAL activity independent of ozone concentration. No modification of ascorbic acid and deshydro ascorbic acid concentration	Rapid increase of the number of bacteria. Growth rate approximately twice that seen on lettuce washed by water. Increase of a* value. Increase of PAL activity	[104]
Polyozone MOD-T-816 generator, oxygen, 9 psi, 1.7 mg L^−1^, 4.6 L min^−1^	60 L of tap water, 10, 20, 30, 40 and 50 min, CT between 13.3 and 17.9 mg min^−1^ L^−1^	300 g of Romaine lettuce artificially contaminated with a suspension of *Bacillus cereus* spores	/	Reduction of *B. cereus* spore concentration by more than 4.4 log CFU g^−1^ in 30 min in water, reduction from 0.95 to 2.08 log on lettuces (an average 1.56 log reduction)	/	/	[105]
Steriline model 1A, compressed air, 3 g h^−1^, 150 L h^−1^	50 L of deionized water, pH = 6.68, 5 min, 1; 2 and 5 ppm,	1 kg of iceberg lettuces shredded into 3 by 3 cm pieces contaminated with *S. sonnei*	/	Reduction of *S. sonnei* counts after 5 min by 0.6, 1.4 and 1.8 log CFU g^−1^ with 1, 2 and 5 ppm	/	/	[106]

**Table 8:** General synthesis of the treatment of salads with ozonated water: advantages and disadvantages.

Treatment Type	Prewashing Treatment	Static Conditions	Dynamic Conditions
Advantages	-Easily implemented in commercial processing lines -Efficient in reducing the microbial load	-Maintains visual and sensorial quality -Efficient in reducing the microbial load -Conservation of nutritional quality	-Maintain visual and sensorial quality -Efficient in reducing the microbial load -Conservation of nutritional quality -Improve quality of water
Disadvantages	-Carried out on whole salads (prior to shredding) to avoid increase of COD in washing water	-Not industrially applicable	-Extreme importance of controlling all processing parameters over time, especially under industrial conditions

**Table 9:** Overview of the impact of gaseous ozone on quality and safety characteristics of tomatoes.

Treatment Conditions	Produce/Targets	Microbial Quality	Physical, Chemical, Nutritional Qualities	References
Glass jars, 5, 10 and 20 mg L^−1^ for 5, 10, 15 and 20 min	Cherry tomatoes (3 cm), *Salmonella enteritidis* onto surface	Reduction of 3 log CFU tomato^−1^ after 10 mg L^−1^ for 5 min and 7 log CFU tomato^−1^ after 15 min at 20 mg L^−1^	A red to yellow change at 30 mg L^−1^, No texture modification.	[115]
Closed chamber with circulating gaseous 0.86 or 1.71 µg O_3_ g^−1^ produce for 2.5 or 5 h at 23 °C	Beefsteak tomatoes *Listeria monocytogenes, Escherichia coli (STEC), Salmonella enterica* 6.5 log CFU g^−1^	Reduction of 1.6 log CFU g^−1^ for *Escherichia*, 1.1 log CFU g^−1^ for *Salmonella* and *Listeria* after 5 h of exposure at 1.71 µg O_3_ g^−1^ produce	Bleaching of the tomato epidermidis if higher concentration and duration used	[70]
Chamber 1.71, 3.43 and 6.85 mg L^−1^ at a flow rate 4 L min^−1^ for 2 or 4 h	Grape tomatoes inoculated on their smooth surface and scar stem with *Salmonella* and native population	Reduction of 2 log CFU fruit^−1^ for *Salmonella* after 6.85 mg L^−1^ concentration for 2 h Reduction of native bacterial population at days 1 and 7 of storage for 3.43 and 6.85 mg L^−1^ concentrations No impact on yeasts and molds	Visual degradation and off-notes aroma after 3.43 mg L^−1^ for 2 h Wet tomatoes suggesting skin rupture after 6.85 mg L^−1^ for 4 h, Only 1/3 of the ascorbic acid was kept at day 21. A progressive Lycopene degradation correlated with red color alteration during storage	[116]
Chamber 800 and 1600 ppm for 30 min coupled or not coupled with hydrogen peroxide	Grape tomatoes inoculated on their smooth surface and scar stem with *Salmonella*	A 0.5 log CFU fruit^−1^ reduction was obtained for ozone gas alone a 5.2 log CFU fruit^−1^ reduction on the smooth surface and a 4.2 log CFU fruit^−1^ on scar stem for ozone gas coupled with aerosolized hydrogen peroxide	/	[117]
0.005 to 1.0 μmol mol^−1^ ozone, at 13 °C and 95% relative humidity	Full-ripe tomatoes 5–6 cm diameter *Botrytis cinerea*	Reduction of spore production/viability of *B. cinerea*	No impact on weight loss, antioxidant status, CO2/H2O exchange, or content of organic acids, total phenol, or vitamin C Management of ripening by ethylene controlling and proteomic changes	[118,119]
chamber 10 min at 20, 35 and 50 ppm	Tomatoes at different stages of ripening 5 cm diameter	Reduction of spoilage	Management of ripening Extension of shelf life of 10 days with a delay of ripening about 3.6 days	[120]
25 or 45 mg m^−3^ for 2 h day^−1^ for 16 days	Green tomatoes	Reduction of spoilage apparition only 14% of damaged fruit versus 54% for the control	Management of ripening No significant impact on pH, titrable acidity, and soluble solids for the two treatments Firmness, weight preservation only with 25 mg m^−3^	[121]
In-package ozone treatment system 1000 ppm for 1, 2 and 3 min	Cherry tomatoes *Listeria innocua, Salmonella* Typhimurium, *Escherichia coli* O157:H7	For *Listeria*: 6 and 3 log CFU unit^−1^ reductions on the smooth part and the scar stem, respectively. For *Salmonella*, 2.7 log and 2.1 CFU unit^−1^ reductions the smooth part and the scar stem, respectively. For *Escherichia,* a decrease of 1.8 to 2.6 CFU fruit^−1^ the smooth part and the scar stem, respectively.	Firmness and color of tomatoes stored 22 days at 22 °C were not noticeably affected by the ozone treatment step in the package	[122]

## Data Availability

The data presented in this study are available on request from the corresponding author.
